# Comparative Physicochemical and Pharmacotechnical Evaluation of Three Topical Gel-Cream Formulations

**DOI:** 10.3390/gels11070532

**Published:** 2025-07-09

**Authors:** Ramona Pârvănescu, Cristina Trandafirescu, Adina Magdalena Musuc, Emma Adriana Ozon, Daniela C. Culita, Raul-Augustin Mitran, Cristina-Ionela Stănciulescu, Codruța Șoica

**Affiliations:** 1 Department of Pharmaceutical Chemistry, “Victor Babes” University of Medicine and Pharmacy, 300041 Timisoara, Romania; ramona.parvanescu@umft.ro (R.P.); trandafirescu.cristina@umft.ro (C.T.); 2 Research Center for Experimental Pharmacology and Drug Design (X-Pharm Design), “Victor Babes” University of Medicine and Pharmacy, Eftimie Murgu Square, No. 2, 300041 Timisoara, Romania; codrutasoica@umft.ro; 3 Institute of Physical Chemistry—Ilie Murgulescu, Romanian Academy, 202 Spl. Independentei, 060021 Bucharest, Romania; dculita@icf.ro (D.C.C.); rmitran@icf.ro (R.-A.M.); 4 Department of Pharmaceutical Technology and Biopharmacy, Faculty of Pharmacy, “Carol Davila” University of Medicine and Pharmacy, 6 Traian Vuia St., 020956 Bucharest, Romania; emma.budura@umfcd.ro; 5 Dermatology Department, Faculty of Medicine, University of Medicine and Pharmacy Craiova, 2 Petru Rares St., 200349 Craiova, Romania; medicine@umfcv.ro; 6 Department of Pharmacology—Pharmacotherapy, “Victor Babes” University of Medicine and Pharmacy, 300041 Timisoara, Romania

**Keywords:** gel-cream formulations, natural cosmetic formulations, oxidative stability, antioxidant capacity, skin barrier function

## Abstract

In the context of modern dermocosmetic development, multifunctional topical gel-cream formulations must be efficient for both therapeutic efficacy and cosmetic applications. This study presents a comparative physicochemical and pharmacotechnical analysis of three topical gel-cream formulations developed by Brand Chanand^®^: Acne Control Cleanser (ACC), Acne Face Cream (AFC), and Gentle Cream Cleanser Serum Control, Regenerating, Hydrating, Calming (IRC). Each formulation is enriched with a specific blend of bioactive compounds, including botanical oils, vitamins, and proteins, designed to treat acne, to support skin regeneration, and to maintain the skin barrier. A multidisciplinary approach was used, including Fourier Transform Infrared Spectroscopy with Attenuated Total Reflectance (FTIR-ATR), differential scanning calorimetry (DSC), rheological evaluation, pH and density determination, spreadability analysis, and oxidative stability testing to evaluate the products. Antioxidant capacity was assessed through multiple in vitro assays. The results demonstrated that all three gel-cream formulations exhibit pseudoplastic rheological behaviour, suitable for topical application. AFC showed the highest oxidative stability and antioxidant activity, while IRC presented superior spreadability and cosmetic efficacy, likely due to its complex composition. ACC displayed faster absorption and was ideal for targeted use on oily or acne-prone skin. The differences observed in the stability and performance suggest that the ingredient synergy, base composition, and solubility profiles show notable variations in dermato-cosmetic formulations. These findings highlight the formulation–performance relationship in topical gel-cream formulations and support the development of new cosmetic products tailored for sensitive and acne-prone skin.

## 1. Introduction

The domain of cosmetic science has evolved significantly in recent years, integrating multidisciplinary research from dermatology, chemistry, materials science, and biotechnology [1,2,3]. Among the most dynamic segments of this field are dermato-cosmetic products, which not only satisfy aesthetic requirements but also offer therapeutic benefits, especially for sensitive and acne-prone skin. Acne vulgaris and its associated conditions are chronic dermatological disorders that affect a wide range of the population, often requiring long-term management through cleansers, moisturizers, and anti-inflammatory formulations [4]. In this context, modern cosmetic creams are expected to exhibit both efficacy and biocompatibility.

Topical formulations such as gel-cream products represent multifunctional compounds that combine active ingredients with carefully engineered bases. These gel-creams are promoted as suitable for sensitive skin and are formulated to perform specific tasks, ranging from cleansing and sebum regulation to skin barrier repair and hydration [5]. Their performance depends not only on the biological activity of the ingredients but also on their physicochemical properties, including pH, viscosity, spreadability, and resistance to oxidative degradation [6,7]. These formulations represent advanced dermato-cosmetic systems based on naturally derived active compounds and are optimized for therapeutic efficacy and skin tolerance [8].

In order to optimize formulation development, a comprehensive evaluation of these products is essential. Analytical techniques such as Fourier Transform Infrared Spectroscopy with Attenuated Total Reflectance (FTIR-ATR), and differential scanning calorimetry provide valuable insights into chemical interactions and thermal behaviour under controlled heating regimes, while rheological testing helps to assess the application and product viscosity behaviour. Additionally, oxidative stability is a crucial parameter for assessing the shelf life and safety of the products.

This study aims to conduct a comparative analysis of three topical gel-cream formulations developed by Brand Chanand^®^ (The products formulations are established by Research & Developed Prop. 65 California Compliant USA and the products are produced in Bucharest, Romania, by Skincores LLC, Bucharest, Romania): Acne Control Cleanser (ACC), Acne Face Cream (AFC), and Gentle Cream Cleanser Serum Control, Regenerating, Hydrating, Calming (IRC) using a combination of physicochemical, pharmacotechnical, oxidative stability evaluations, antioxidant capacity and in vivo investigations of the cosmetic products behavior. By integrating results from multiple methodologies, the research aims to better understand how formulation characteristics influence the product performance and the stability related to their final scope. Such an integrated approach is essential for controlling future developments in the dermato-cosmetic formulations and ensuring advancement in the cream-based gel formulation design.

## 2. Results and Discussion

### 2.1. Organoleptic Properties

The organoleptic evaluation of the three gel-cream formulations—ACC, AFC, and IRC—was performed by direct observation and tactile assessment under standardized laboratory conditions [9,10]. The results are shown in Table 1.

### 2.2. Density and pH Determination

The density and pH values of the studied creams (ACC, AFC, and IRC) were determined and are presented in Table 2. From Table 2, a difference in density was observed between AFC (0.99 g/cm^3^) and ACC (0.62 g/cm^3^) formulations. This is attributed to the distinct compositions. AFC, which is a semisolid cream, contains a higher proportion of lipid-based ingredients, whereas ACC, being a cleanser, includes more water and surfactants, resulting in a lower bulk density with a fluid gel-cream formulation.

All three gel-cream formulations exhibited a slightly acidic pH, which falls within the physiological pH range of human skin (4.5–6.5). This is favourable for maintaining the integrity of the skin barrier, supporting the microbiome, and minimizing the risk of irritation. The differences in density among the formulations reflect their varying compositions and textures, with AFC showing the highest density, consistent with its more compact consistency.

### 2.3. Spreadability Assessment

**Figure 1:**
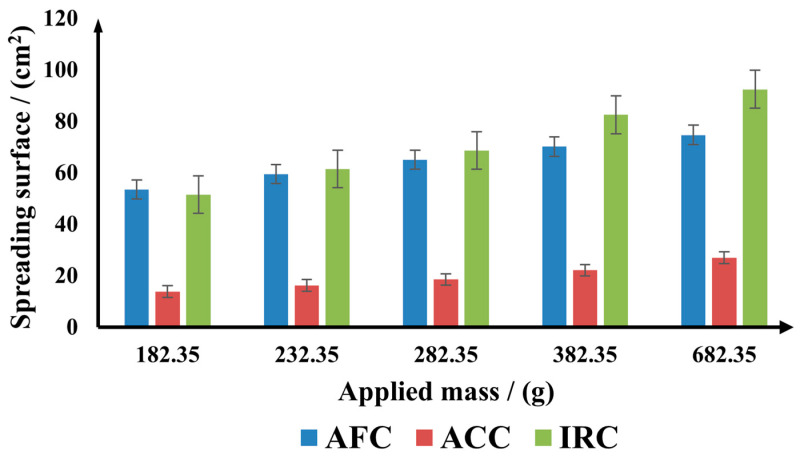
The spreading properties of the studied gel-cream formulations.

The spreadability of the three gel-cream formulations was evaluated using the extensometric method, with results recorded for five weights weighing 182.35 g, 232.35 g, 282.35 g, 382.35 g, and 682.35 g. The results are given in Figure 1.

The data from Figure 1 revealed notable differences among the products:

(i) ACC showed the lowest spreadability values. This suggests a lower spreading performance compared to the other two gel-cream formulations. The ACC formulation was designed to have a higher viscosity, allowing for a prolonged contact time. Such a profile is advantageous for localized treatments, oily or acne-prone skin, where quick absorption is preferred, even though the spreadability is lower, as the gel-cream formulation was designed.

(ii) AFC is classified second in terms of spreadability, showing a consistent increase in spreading surface throughout the evaluation, although it remained below IRC. Its profile indicates a balanced formulation, offering both good spreadability and effective absorption. This makes it suitable for general daily use, especially for individuals with normal to combination skin types.

(iii) IRC exhibited the highest spreadability values throughout the entire applied mass, increasing progressively. This suggests that IRC has the greatest spreading capacity, potentially allowing it to cover a larger surface area with a smaller amount of product. Its high spreadability implies slower absorption, making it suitable for extended hydration and barrier function. As such, IRC is particularly appropriate for dry or very dry skin, and its use may be especially suitable as a night-time treatment.

These differences in spreadability align with the intended therapeutic goals and targeted skin types of each product, highlighting the importance of rheological and sensory characteristics in topical gel-cream formulations.

### 2.4. Oxidative Stability Assessment

The oxidative stability of the studied gel-cream formulations was assessed by monitoring the variation in oxygen pressure during accelerated oxidation. The obtained pressure curves reflect the consumption of oxygen as lipid oxidation progresses. A characteristic inflection point on the curve indicates the product’s intrinsic resistance to oxidation, marking the onset of accelerated oxygen uptake that correlates with the initial formation of rancid odors and degradation products. The induction period (IP), determined by the intersection of tangent lines drawn before and after the inflection point, was used for the quantitative measure of the oxidative stability. A longer IP indicates a greater resistance to lipid peroxidation and a longer shelf life under oxidative stress. The results are presented in Table 3.

According to Table 3, among the tested gel-cream formulations, AFC demonstrated the highest IP, signifying superior oxidative stability. This is attributed to its composition of highly stable ingredients and a robust antioxidant profile, which effectively protects against oxidative degradation.

In contrast, ACC contains a higher proportion of vegetable oils rich in unsaturated fatty acids, such as *Abyssinian seed oil* and *Mango seed* butter, which, despite their nourishing properties, are more vulnerable to oxidation. The inclusion of vitamin E in ACC provides partial antioxidant protection; however, it is insufficient to fully mitigate the susceptibility of these oils to lipid peroxidation. This results in a moderately lower IP compared to the AFC.

The IRC formulation, while designed for enhanced cosmetic efficacy through a complex blend of active ingredients, lacks a comprehensive antioxidant system. Consequently, it exhibited the shortest induction period, indicating a higher tendency for oxidative degradation under the test conditions.

These findings highlight the essential role of each gel-cream formulation composition in oxidative stability, with implications for product shelf life, packaging choices, and recommended storage conditions. Formulations with longer IPs, such as AFC, are expected to maintain their efficacy and sensory qualities over extended periods, whereas those with lower IPs, like IRC, may require additional antioxidant fortification or protective packaging to ensure product integrity.

### 2.5. Fourier Transformed Infrared Spectroscopy—Attenuated Total Reflectance (FTIR-ATR) Analysis

**Figure 2:**
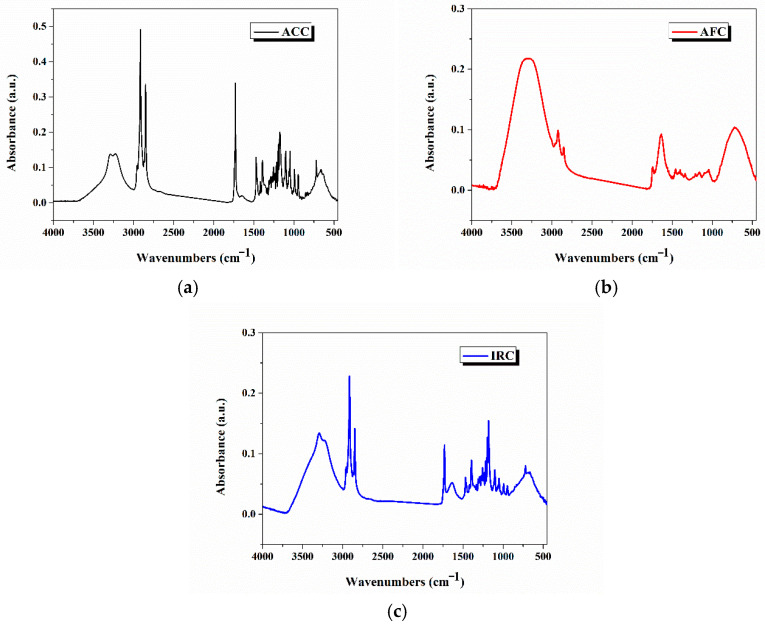
The FTIR-ATR spectra of (**a**) ACC, (**b**) AFC, and (**c**) IRC.

The FTIR-ATR spectra of the three analysed samples are shown in Figure 2a ACC, (b) AFC, and (c) IRC, and were used to characterize the gel-cream formulations based on the mentioned active components presented in Table S1 in the Supplementary Materials.

The main FTIR-ATR peaks of the ACC formulation (Figure 2a) are represented in Table S2 from the Supplementary Materials. The FTIR-ATR spectrum of the ACC nourishing formulation (Figure 2a) reveals the following bands: the broad band resulted from the overlapping of O-H and N-H bands of stretching vibration centered at 3291.9 cm^−1^ and 3226.3 cm^−1^, which confirms the presence of alcohols, carboxylic acids, or amines, matching well with hydrating agents like *Abyssinian seed* oil, as it is mentioned in the ACC composition. Strong aliphatic symmetric and asymmetric C–H stretching vibration bands, which appear at 2914.9 cm^−1^ and 2849.3 cm^−1^, support the presence of oils and fatty compounds (from *Abyssinian seed* oil) in the ACC composition. Carbonyl functional bands (C=O stretching vibration), which appear near 1729.8 and 1640 cm^−1^, are indicative of esters (from butters/oils) and amides (from rice proteins). The amide I band at 1640 cm^−1^ supports the presence of protein-based ingredients such as rice protein. The two absorption bands at 1469.5 cm^−1^ and 1392.4 cm^−1^ are assigned to CH_2_ and CH_3_ bending vibrations of the ethyl and methyl groups. The C–O stretching vibrations of ester bonds and C–N stretching vibrations between 1300 and 1050 cm^−1^ suggest the presence of esters, alcohols, and possibly sugar derivatives from natural extracts [11,12].

The main FTIR-ATR peaks of the AFC anti-acne cream formulation (Figure 2b) are represented in Table S3 from the Supplementary Materials. The FTIR-ATR spectrum of the AFC anti-acne cream formulation (Figure 2b) reflects the presence and the synergy between hydrating active ingredients (betaine), lipid-replenishing agents (phytosterol esters), biological extracts (*Chlorella* extract), and antioxidants (vitamin E) [13,14,15].

The main FTIR-ATR peaks of the IRC intensive repair complex cream cleanser formulation (Figure 2c) are represented in Table S4 from the Supplementary Materials. The FTIR-ATR spectrum of IRC formulation confirms the strong moisturizing and barrier-repair properties (due to the presence of functional groups O–H, C–O, amide from the panthenol, niacinamide, and betaine compounds), the presence of lipids and antioxidants (based on the presence of C–H stretches, ester C=O, esters from mango seed butter, the anti-inflammatory and soothing actives (based on the presence of the bands which correspond to niacinamide and panthenol compounds) and the presence of the mild protein-based cleansing agents (from rice protein, algae extract) based on the presence of the amide I (1636.3 cm^−1^) band [16,17]. The spectrum from Figure 2c is fully consistent with the stated main composition of the IRC formulation.

The FTIR-ATR spectral analyses of the ACC, AFC, and IRC formulations confirm the presence of the most important functional groups that correspond to most of the specified active ingredients.

### 2.6. Differential Scanning Calorimetry (DSC)

**Figure 3:**
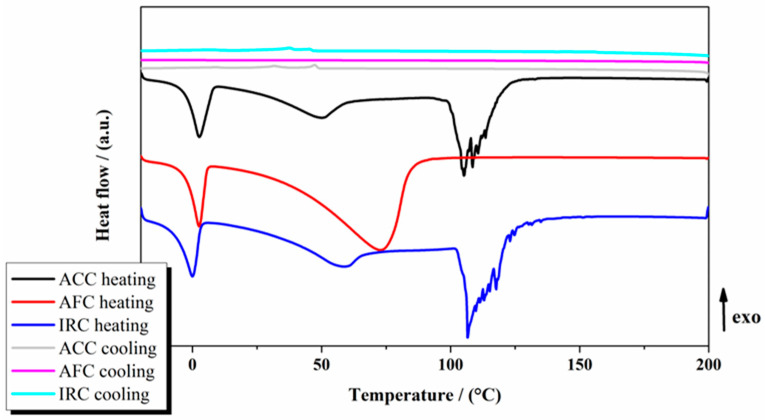
DSC analyses of the studied gel-cream formulations.

DSC analyses of the samples were carried out for all samples between −20 and 200 °C, at a temperature change rate of 10 °C min^−1^ (Figure 3).

**Figure 4:**
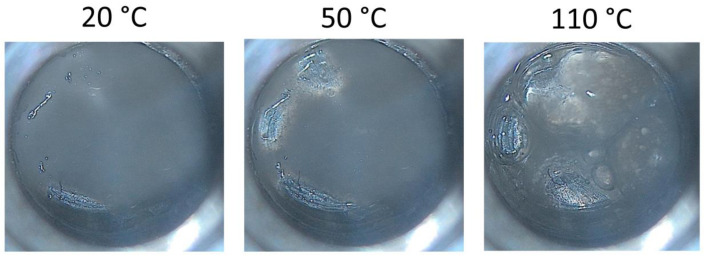
Representative optical microscopy images of the ACC sample at different temperatures (20 °C, 50 °C, and 110 °C).

Water melting can be noticed for all samples during the heating cycle. An endothermic transition between 20 and 70 °C can also be noticed. This transition represents the melting of the organic components. Finally, water evaporation occurs after 100 °C for the ACC and IRC samples. Water evaporation seems to overlap with the organic melting for the AFC sample. The DSC events were confirmed by optical microscopy (Figure 4), exemplified for ACC formulation. The sample’s melting and water evaporation can be noticed. In Figure 4, the melting process is indicated by the visible transition from a solid-like structure to a more fluid, less defined morphology, as observed under thermal microscopy during controlled heating. The water evaporation is suggested by the formation of microbubbles and gradual reduction in sample opacity, which occur concurrently with temperature increase.

The organic melting temperature varies between 29.8 °C and 40.4 °C. The water content was computed from the heat of fusion values, and it varies between 25.5 and 58.1%wt. (Table 4).

### 2.7. Rheology Analysis

Rheology method represents an important step in evaluating the practical handling and performance of topical dermocosmetic creams during various stages of their usage, including application, mixing, storage, and administration [18]. The flow and deformation behaviour of these formulations directly influence their spreadability on the skin, their maintained homogeneity when mixed or blended, and their stability during storage to prevent phase separation. Additionally, rheological properties affect the efficiency and consistency of pumping or dosing mechanisms used in manufacturing and packaging. Understanding and optimizing the rheological characteristics is consequently essential to ensure user-friendly products that bring the intended scope while maintaining physical stability and aesthetic, cosmetically appealing throughout their use [19,20]. The obtained dynamic viscosity values were used to assess the flow behaviour of the formulations, with a focus on non-Newtonian characteristics and structural recovery after shear stress, which are important properties for the stability and performance of topical dermocosmetic products.

**Figure 5:**
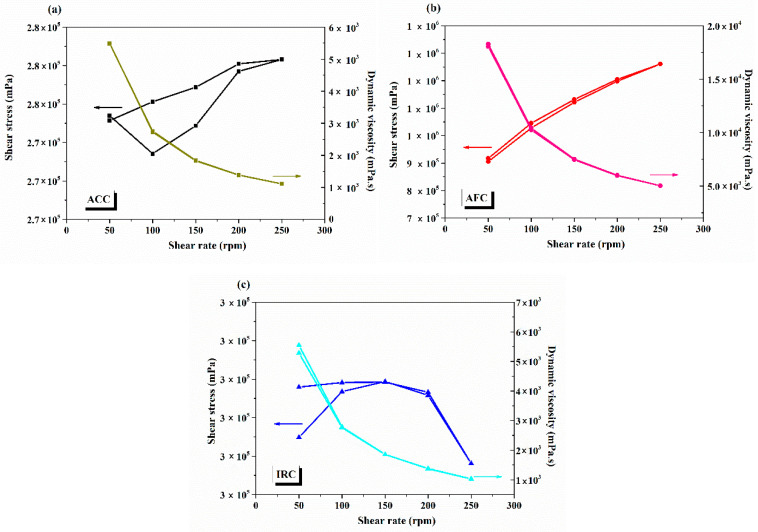
Variation in dynamic viscosity and shear stress as a function of shear rates in (**a**) ACC, (**b**) AFC, and (**c**) IRC.

Figure 5a–c shows the graphical representation of data obtained from rheology measurements.

From Figure 5, it was observed that all three gel-cream formulations exhibit a pseudoplastic (shear-thinning) rheological behaviour, as the viscosity shows a decrease as shear rate increases throughout the entire examined shear rate range, which is highly required for topical applications. These formulations remain relatively viscous or semisolid at rest, ensuring the product’s stability and the ease of handling, but become more fluid under shear stress, facilitating easy dispersion during application on the skin. ACC and IRC presented lower viscosity values and smoother flow profiles, revealing lighter textures. These properties contribute to easier application and rapid absorption during usage, and they represent essential characteristics that are particularly beneficial for cleansers and repair creams intended for daily or frequent use. Contrary to ACC and IRC formulations, AFC has a higher viscosity, suggesting a denser and more structured consistency. This increased resistance to flow may enhance the occlusive effects by creating a protective barrier on the skin surface to minimize and prevent water evaporation, providing a prolonged contact with the skin, and increasing the efficacy of its active anti-acne compounds. These rheological differences between the three formulations reflect not only the compositional differences between them but also their intended dermatological use.

### 2.8. Antioxidant Activity

#### 2.8.1. DPPH Radical Scavenging Activity

**Figure 6:**
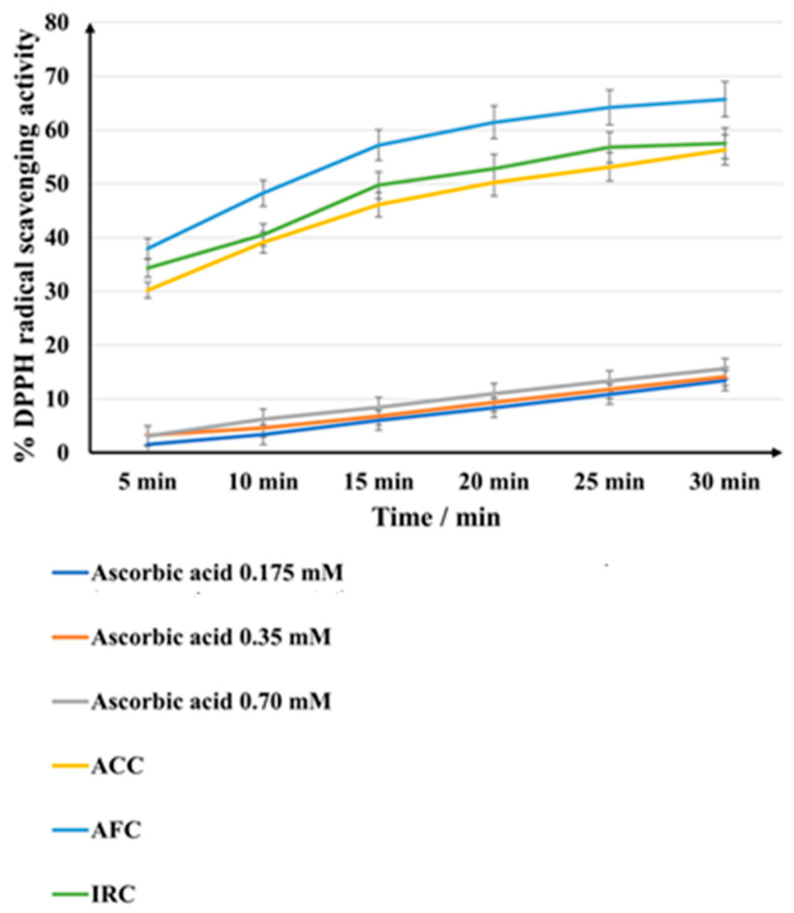
DPPH radical scavenging activities were exhibited by the samples and the positive controls.

Figure 6 presents the percentage of DPPH radical scavenging activities exhibited by the samples and the positive controls (vitamin C 0.175 mM, 0.35 mM, and 0.7 mM).

All cosmetic products outperformed the reference ascorbic acid, which is considered to have strong antioxidant properties. The highest scavenging of DPPH radicals (up to 65.72% after 30 min) was observed for AFC cream. ACC and IRC showed quite similar performances, with the IRC formulation showing a slightly higher antioxidant effect (57.53% after 30 min). It should be noted that all three products exhibited a higher electron donor capacity after the first 5 min than any of the ascorbic acid samples used as a reference. The RSA percentage increases gradually over time in all samples, increasing more rapidly in the first 20 min, after which the rise is maintained, but the differences between 20 and 30 min are not marked.

#### 2.8.2. ABTS Radical Scavenging Activity

**Figure 7:**
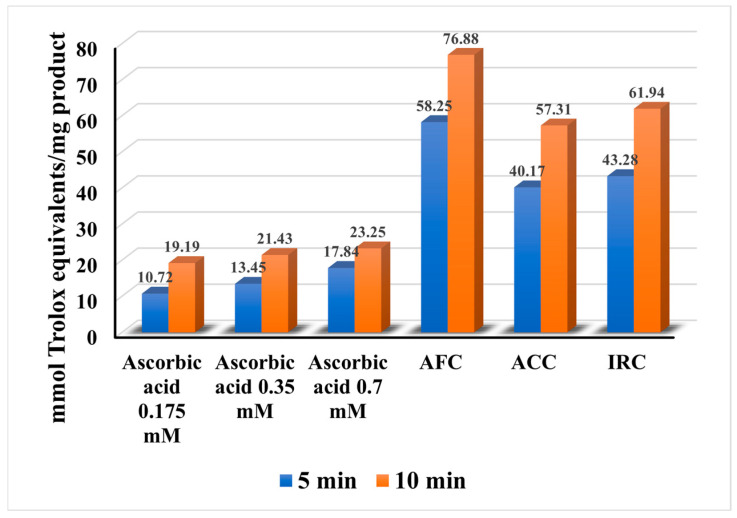
ABTS radical scavenging abilities of the samples and the positive controls.

The ABTS radical scavenging ability of the samples is presented in Figure 7.

In agreement with the data obtained for the DPPH scavenging effect, the samples showed a similar behavior. For ascorbic acid, the performance gradually increases with concentration, and for the cosmetic products, AFC still shows the highest antioxidant efficacy, both after 5 and 10 min, with an inhibition of 58.25% and 76.88%, respectively. However, the results show that all products, regardless of their physical system or composition, have a higher radical scavenging activity than ascorbic acid, as a control. As in the DPPH analysis, the lowest ABTS scavenging activity of the cosmetics was observed for ACC with 40.17% after 5 min and 57.31% after 10 min.

#### 2.8.3. FRAP Assay

**Figure 8:**
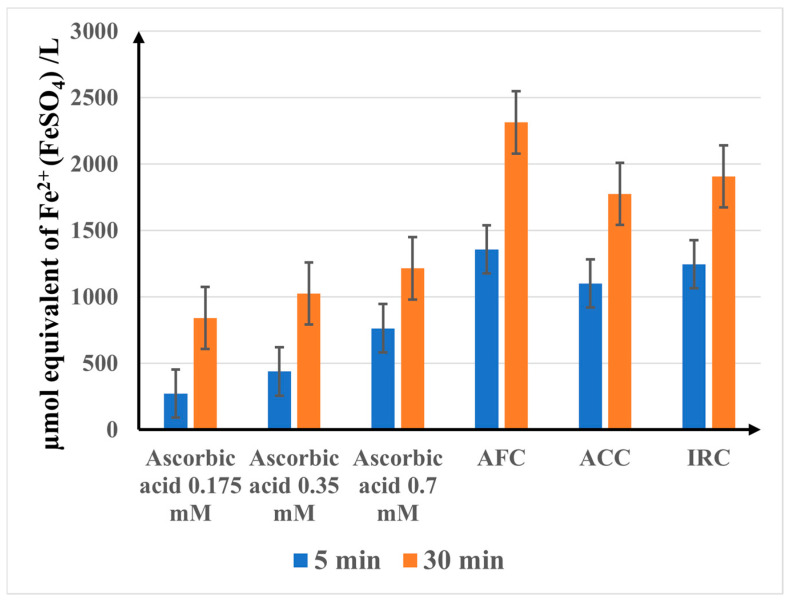
FRAP assay results.

The FRAP values of the samples (µmol equivalent of Fe^2+^ (FeSO_4_)/L) recorded at 5 and 30 min are shown in Figure 8.

The results of the FRAP assay are consistent with the findings of the other two in vitro antioxidant capacity studies, with the cosmetic products showing a much higher efficiency than the ascorbic acid reference solutions. As found in the previous analyses, the antioxidant ability of the tested products increases in the order IRC < ACC < AFC.

According to the product’s labels, several compounds with known antioxidant properties were included in the formulations. In particular, all three tested products contain vitamin E, which possesses a well-known high antioxidant function [21] mainly by protecting lipophilic compounds from oxidative damage [22]. However, the antioxidant mechanism of vitamin E is to scavenge free lipid peroxyl radicals, which are by-products of the chain reaction of lipid peroxidation. This mechanism is most effective in protecting the lipid membranes of the cell, and vitamin E is abundant [23].

The results of the in vitro analyses to prove the antioxidant capacity of the products have shown that the anti-acne cream (AFC) has the highest potential to inhibit reactive oxygen species (ROS) for all three methods. It can be assumed that the addition of *Chlorella vulgaris* extract and phytosterol esters from *Crambe abyssinica* may contribute to the antioxidant capacity of the cream, probably through a synergetic effect. Adhoni et al. [24] revealed that *Chlorella vulgaris* contains a large amount of flavonoids and phenols, two secondary metabolites with antioxidant qualities. Putri et al. [25] also found a high proportion of fatty acids in the phytochemical composition of the green microalgae, which can act as antioxidant molecules [26]. Flavonoids can prevent lipid oxidation and scavenge free radicals [27]. Phenolic compounds can donate hydrogen, and their antioxidant effect can be achieved either at the end of the radical chain reactions or during the neutralization reaction of the free radicals that start the oxidation process. The amount of hydroxyl groups in the chemical structure determines the antioxidant performance of phenolic and flavonoid components [26]. Wu et al. [28] discovered a significant content of β-carotene, ascorbic acid, and tocopherols, molecules with known antioxidant properties, in the chemical composition of *Chlorella vulgaris* in addition to the compounds mentioned above. On the other hand, Siti et al. [29] demonstrated that chlorophyll is the bioactive compound responsible for the antioxidant properties of *Chlorella vulgaris* extract. The authors carried out the analyses on different cosmetic products, varying various factors such as the amount of extract, the methods for its incorporation in the base, or the type of product. The results led to the conclusion that only by increasing the chlorophyll content can the antioxidant capacity be enhanced, regardless of the other technological factors. According to Chatzikonstantinou et al. [30] findings, of all Chlorella strains, C. vulgaris proved to exhibit the greatest antioxidant activity.

Regarding the phytosterol esters from *Crambe abyssinica*, Iwassa et al. [31] reported that their significant antioxidant efficiency is mainly due to the content of β-sitosterol. According to González-Larena et al. [32], the antioxidant ability of the crambe seeds’ phytosterols increases with the β-sitosterol concentration.

The AFC product also contains betaine, whose antioxidant effect is the subject of intensive research, although the theories about its mechanism of action are controversial. While Alirezaei et al. [33,34] have conducted several studies suggesting that the antioxidant capacity of betaine is due to the increase in antioxidase activity, Balkan et al. [35] have proven that there is no correlation between these. The study conducted by Erman et al. [36] showed that betaine increases the activity of SOD (Superoxide Dismutase). Zhang et al. [37] conducted an important study and concluded that the three methyl groups of betaine are essential for its antioxidant activity and found that the mechanism is based on the enhancement in nonenzymatic antioxidant defenses by forming a protective membrane around the cells.

The results of the investigations on the antioxidant potency of AFC in the present study revealed its high performance, and it is assumed that this is due to the additional effect of the components, as they can rely on multiple studies that prove their individual efficiency.

It is interesting to note that both ACC and IRC have a lower antioxidant effect than AFC, although they contain crambe seed oil, which, in addition to phytosterol esters, also contains high amounts of tocopherols, especially γ-tocopherol, which is considered a stronger antioxidant than α-tocopherol [38]. There are several explanations for this: the oily phase, which acts more slowly, the inhibitory effect of other ingredients in the products, or the low concentration of oil in the formulations.

In addition, both products (ACC and IRC) contain rice proteins and mango butter, both of which have been shown to have an intense antioxidant effect. The results of the study by Chen et al. [39] suggest that rice proteins have strong ABTS radical scavenging activity and also antihyaluronidase and antityrosinase activity, which is beneficial for cosmetic applications. As found in several recent studies [40,41], the hydrogen donor activity of the aromatic amino acid hydroxyl groups contributes to their high capacity for scavenging radicals, and certain side-chain groups or the particular peptide structure can donate protons or electrons.

Various studies have attributed the antioxidant effect of mango butter to different ingredients. Nunez-Selles et al. [42] proved that it includes stable fats with a high proportion of saturated fatty acids, phospholipids, and various phenolic substances, and they assume a synergistic effect of these substances. Ahmed et al. [43] found that tocopherols and carotenoids may also be involved in the antioxidant activity of mango seed butter. According to the study of Y. Y. Soong and P. J. Barlow [44], mango butter possesses a high phenolic and tocopherols content, a high source of phytosterols such as stigmasterol, β-sitosterol, and campesterol, and has a strong antioxidant activity. Maldonado-Celis et al. [45] found that the antioxidant ability of mango butter is due to the high content of polyphenols, phytosterols, sesquiterpenoids, and microelements like selenium, copper, and zinc.

In addition to these ingredients, IRC also contains other effective antioxidant ingredients: niacinamide, cranberry oil, betaine, and panthenol.

Zhen et al. [46] demonstrated that niacinamide inhibits particulate matter 2.5 (PM2.5)-induced ROS generation and interrupts PM2.5-induced oxidation of various molecules, such as lipids, proteins, and DNA. Abdullah et al. [47] also found that niacinamide significantly suppresses oxidative stress in vivo and supports the repair of damaged DNA.

Cranberry oil has a high concentration of bioactive compounds, especially flavonoids, which are known to have antioxidant properties [48]. It also contains anthocyanins, which have anti-inflammatory and antioxidant qualities through their aglycones and anthocyanidins. Proanthocyanidins, flavonols, primarily quercetin and myricetin glycosides, and triterpenoids, of which ursolic acid is the predominant one, are also abundant in cranberries [49]. It has been shown to possess antioxidant qualities in cancer cell lines [50].

Betaine is a naturally occurring substance that has been extensively researched as an antioxidant in human health, but its antioxidant mechanism is still unknown. According to an interesting and extensive study developed by Zhang et al. [51], betaine exhibited minimal free radical scavenging action, according to radical scavenging assays. However, erythrocyte hemolysis and cellular antioxidant activity (CAA) tests ascertained betaine’s antioxidant potential. The antioxidant activity of betaine was not caused by the gene expression and activity of antioxidases, according to the results of quantitative PCR and enzyme activity detection kits. By controlling the methionine–homocysteine cycle, betaine raised the amounts of nonenzymatic antioxidants, S-adenosylmethionine (SAM), and methionine, according to an analysis of its impact on the metabolism of sulfur-containing amino acids using high-pressure liquid chromatography. Furthermore, it was discovered that betaine’s three methyl groups are crucial to its antioxidant properties. The potential explanation was that a strong protective membrane was created around cells to stop oxidative stressors from causing ROS production and cell damage due to the hydrophilicity of betaine’s carboxyl and the hydrophobicity of its three methyl groups. The antioxidant mechanism of betaine was discovered to improve nonenzymatic antioxidant defenses through the formation of a protective layer around cells and the methionine–homocysteine cycle.

The body quickly oxidizes panthenol, a naturally occurring amine-containing diol, to pantothenic acid, or vitamin B5. Research showing that panthenol has moisturizing and anti-inflammatory qualities when applied to the skin supports its use in cosmetic formulations [52]. In an extensive study, Wang et al. [53] demonstrated that panthenol possesses proangiogenic, antibacterial, anti-inflammatory, and antioxidant qualities that can restore the mechanical and electrophysiological characteristics of skin and hasten wound closure.

The results show that IRC has better efficacy than ACC, which is probably due to the additional ingredients. However, IRC revealed a weaker antioxidant capacity than AFC in all three analyses, which cannot be explained by the number of compounds contained. The differences in the antioxidant performance of the three cosmetic products may be due to a synergistic effect between the ingredients, to the base used, or to the type of cosmeceutical form, or even to the different solubility of the ingredients in various solvents.

### 2.9. In Vivo Investigations of the Cosmetic Products’ Behavior

#### 2.9.1. pH on the Skin Surface

**Figure 9:**
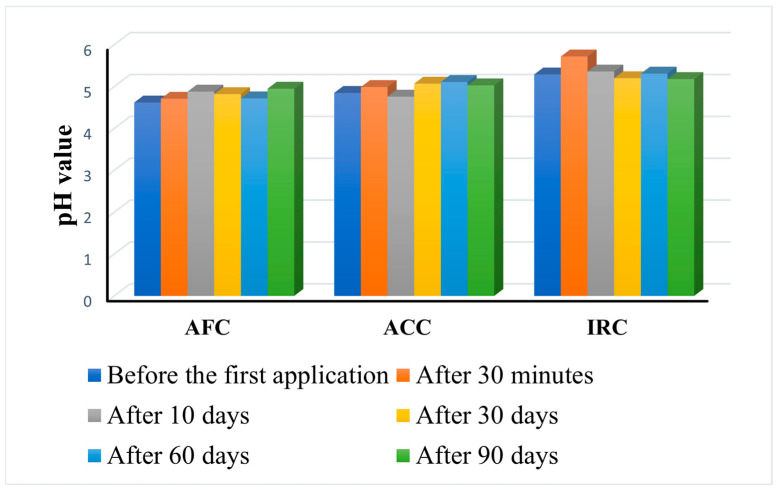
Skin pH measurements.

The human skin not only serves as a barrier against external infections, but also harbors a diverse microbiome that includes viruses, fungi, bacteria, and archaea. Disorders of the skin microbiome can impair immune function and lead to autoimmune and inflammatory diseases. The pH value is a key characteristic of the skin for the microbiome. Cosmetic skincare products are essential for maintaining the microbial balance as they interact with the skin’s pH and microbiome. Products with unphysiological pH levels can disrupt the skin microbiome. A combination electrode, consisting of an H^+^ ion-sensitive electrode and an additional reference electrode, is the basis for the Skin-pH-Meter PH 905 measurements. The results of the skin pH measurements are represented in Figure 9.

As an obvious conclusion, none of the products alter the pH of the skin, which means that they are well tolerated even with long-term use.

However, the behavior of the products is different for the three formulations. AFC gradually increases the skin pH to the highest recorded value after 3 months of daily use (4.95 ± 0.31 compared to 4.62 ± 0.53 before use). ACC also increases the skin pH, but the highest value (5.11 ± 0.54) was measured after 60 days of constant use, while it decreases slightly after 90 days (5.03 ± 0.60). IRC manifests differently, leading to a remarkable increase in pH 30 min after the first application (5.72 ± 0.67 compared to 5.29 ± 0.84 before the first application), but the value slowly decrease over time inducing, after 90 days of application, a final pH value lower (5.18 ± 0.71) than before application.

Although there were some differences in skin pH between volunteers, with subject no. 17 (age 29) having the lowest pH prior to application (4.4), and subject no. 3 (age 47), having the highest pH value at the beginning of the study (5.4), the trend was similar during the study period. Considering that IRC has the lowest pH (5.05) of all three products, its manifestation after application on the skin is not surprising. Nevertheless, the change in skin pH is not linear for any of the products. There are variations between different time intervals, but it is significant that there are no differences in responses between subjects.

In summary, an increased variety of the natural skin pH was observed during the course of the investigation. According to the Shannon diversity index [54], none of the test products significantly alter the diversity of the skin microbiome. Janssens-Böcker et al. [55] proved that using low-pH skincare products does not alter the diversity of the skin microbiota and may even improve skin microbiome diversity and health by lowering the numbers of some harmful bacteria. The acidic pH of the stratum corneum appears to play an important role both in the antimicrobial defense of the skin and in the formation of the permeability barrier [56]. The pH of the skin changes greatly throughout the stratum corneum, which presumably plays a role in regulating enzymatic activity and skin renewal [57]. Numerous endogenous factors, including skin moisture, perspiration, sebum, anatomic location, genetic predisposition, and age, influence the pH of the skin [58]. Variations in pH have been linked to the etiology of skin conditions such as irritant contact dermatitis, atopic dermatitis, ichthyosis, acne vulgaris, and Candida albicans infections [59]. According to Schmid-Wendtner et al. [60] findings, the prevention and treatment of certain skin conditions may benefit from the use of skin products with a pH of roughly 6.

#### 2.9.2. Hydration Level

Improving skin hydration can lower sebum production, enhance the function of the skin barrier, and lower the risk of hyperkeratinization and colonization by microorganisms. Thus, topical anti-acne products with moisturizing properties may be more useful for the treatment of acne, particularly in individuals with dry, irritated skin. In addition, a more moisturizing formulation leads to better adherence to the treatment [61]. Corneometry is currently one of the most widely used methods in the field of dermocosmetics. The experts of the EEMCO (European group on efficacy measurement and evaluation of cosmetics and other products) expressly recommend the Corneometer^®^ (Cologne, Germany) for testing the moisture content of the skin [62].

The stratum corneum, the uppermost layer of the skin, is used as a dielectric medium in the analysis based on capacitance measurements. Its dielectric properties change with increasing water content. Water has a higher dielectric constant (81) than most other substances (usually <7), being the basis for the measurement [63]. When Hua et al. compared Courage + Khazaka corneometry with other devices, they found that while the results for certain skin attributes were related to the measurements of other devices, the results for skin hydration and TEWL showed minimal variability [64]. According to Holm et al. [65], corneometry is a useful non-invasive tool for measuring the severity of atopic eczema in patients.

**Figure 10:**
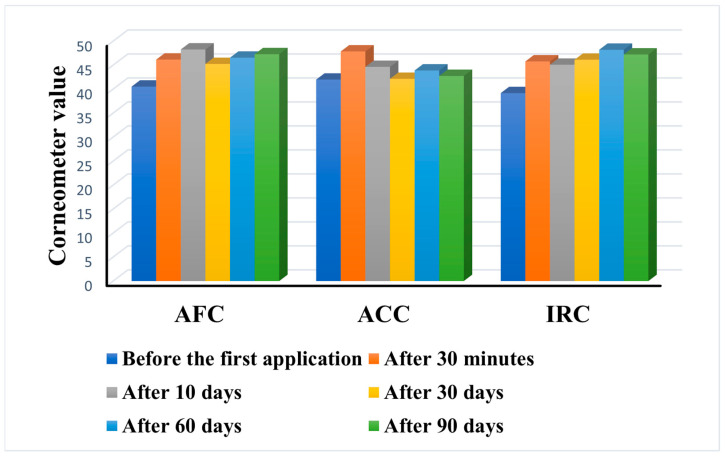
Skin hydration level.

The results of the skin hydration levels are presented in Figure 10.

All three products were found to notably increase the skin’s moisture content after a single application. Even after three months of daily use, all three formulations were found to be moisturizing, but the performance of each product was different. The highest increase in skin hydration was achieved by IRC with a value of 47.22 ± 2.02 after 90 days, compared to 39.17 ± 4.18 before the first application. A similar behavior was also observed for AFC, with a final value of 47.28 ± 1.7 compared to 40.52 ± 2.47 at the beginning. ACC showed the weakest effect with a slight increase from 41.98 ± 3.25 to 42.74 ± 2.59 after 90 days. While AFC and IRC led to a constant increase in hydration over time, AFC showed a fluctuating behavior post-application.

In addition, the performance of the products in six of the subjects differed from the mean behavior registered in the other participants. For subject no. 5 (age 38), only AFC led to an increase in skin hydration, while IRC and ACC had no effect on the parameter. Subject no. 17 (age 29) showed a significant decrease in hydration level after 90 days of ACC application (from 48.06 to 42.13). Subject no. 20 (age 50) showed no change in hydration level at any time following ACC and IRC application. Subject no. 22 (age 27) and subject no. 34 (age 25) showed a significant increase in hydration after the application of ACC. The 90-day application of IRC in subject no. 36 (age 27) resulted in a slight decrease in skin hydration.

The moisturizing ability of the three products can be explained by their components. In AFC, the extract of *Chlorella vulgaris* and the phytosterol esters from *Crambe abyssinica* in particular help to retain moisture in the skin due to the content of amino acids, vitamins, and fatty acids, which leads to increased hydration [66]. IRC also contains *Crambe abyssinica* oil and betaine, which are known for their moisturizing effects [67]. Meanwhile, ACC also contains a large amount of *Crambe abyssinica* oil.

Skin damage caused by excessive dryness or oiliness can be prevented by moisturizing the skin, which also helps to prevent common skin conditions such as acne. In addition to protecting the skin barrier and reducing the formation of excess oil that can clog pores and lead to further acne breakouts, a moisturizing agent also helps maintain adequate moisture levels [68]. The use of moisturizers as supportive therapy has been shown to improve the treatment of some dermatological conditions [69].

#### 2.9.3. Melanin Content

**Figure 11:**
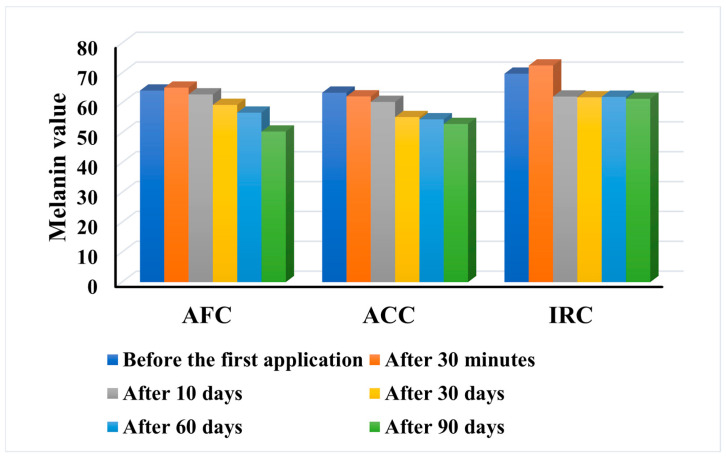
Melanin content.

An important factor in the development of acne is inflammation, which causes capillary hyperplasia, prolonged vasodilation, and aberrant melanin synthesis, which in turn leads to postinflammatory erythema (PIE) and postinflammatory hyperpigmentation (PIH). While PIH is more common in people with darker complexions, PIE mainly affects people with lighter skin tones [70]. A significant percentage of acne patients experience PIH over an extended period of time, which emphasises the need for effective treatment methods. Therefore, anti-acne products should have effective properties to reduce pigmentation alterations. The results of the melanin content are presented in Figure 11.

The results indicate that all three tested products lead to a reduction in the melanin content of the skin after prolonged use. However, the behaviour of the products is different. While AFC and IRC show a slight increase in the melanin value 30 min after the first application, only ACC leads to a decrease in the parameter after the first application and maintains this decrease constant over time, from 63.44 to 52.96. Nevertheless, the greatest decrease in melanin was recorded after the application of AFC, with a decrease in the median value from 64.15 before the test to 50.42 after three months of daily application. The same pattern as with the AFC was observed with the IRC, but the decrease was much smaller in this case, from 69.8 to 61.39. However, in two subjects (no. 22—age 27 and no. 17—age 29), the melanin content was not affected by IRC, with final values similar to the initial values, but they responded with a reduction after the application of ACC and AFC. Also, one subject (no. 38—age 46) showed an increase in melanin content after the application of ACC (from 59.84 to 62.16), but a decrease after the application of AFC and IRC. It is important to note that in all subjects, the melanin content decreased after the continuous application of ACC and AFC, and only in 9 out of 40 (22.5%), the parameter also decreased after the first application of AFC. In the remaining 31 subjects (77.5%), the melanin content increased slightly but decreased again after long-term use.

The results demonstrate the positive effect of all three products on reducing hyperpigmentation of the skin, with AFC showing the highest efficacy, but ACC showing a constant behavior.

*Chlorella vulgaris* extracts have been found to suppress melanogenesis by inhibiting tyrosinase, a key enzyme in melanin synthesis [71]. In addition, Cho et al. demonstrated that betaine reduces the amount of melanin in the cells by directly inhibiting the activity of the tyrosinase enzyme and downregulating MITF at both the transcriptional and post-translational levels. Three signaling pathways seem to be involved simultaneously: activation of the ERK signaling pathway, AKT-GSK3β activation, and PKA-CREB repression [72]. Moreover, the study conducted by Kamei et al. [73] proved that vitamin E suppresses the activity of tyrosinase, a crucial cascade enzyme involved in melanin synthesis in melanoma cells, by 34%, apart from the 28% inhibition of melanin synthesis in B16 cells. According to the results of the present study, the combination of these ingredients in the AFC formulation appears to lead to increased efficacy in reducing the melanin content of the skin. According to the study by Kim H-K [74], melanin production in HRM-2 mice was significantly and strikingly reduced by the radish extract. Additionally, the radish extract cream showed superior skin-whitening and anti-wrinkle properties in humans, demonstrating its strong whitening effects. Its presence in the ACC formulation probably gave rise to its constant and pronounced anti-melanogenic activity. On the other hand, IRC contains niacinamide that can both enhance PIH and decrease melanosome transfer from melanocytes to adjacent keratinocytes [75].

#### 2.9.4. Degree of Erythema

**Figure 12:**
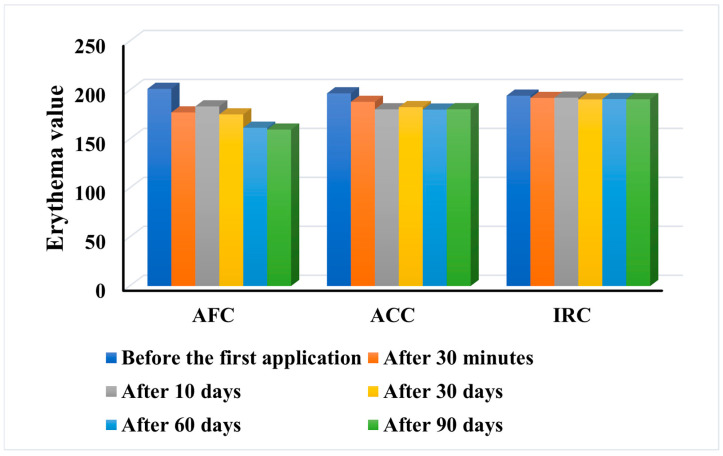
Erythema index.

Skin erythema is one of the most common symptoms of acne, and following an inflammatory reaction, angiogenesis leads to acne-related erythema [76]. One of the aims of dermatocosmetic anti-acne products is to reduce erythema. The degree of erythema after application of a skin care product is also a measure of its tolerability and is recommended to be assessed for each new formulation developed. The results of the erythema index are presented in Figure 12.

It was found that all products diminished the degree of erythema in most subjects, resulting in a decreased mean haemoglobin index value. The most obvious decrease in erythema degree was reported for AFC, which reduced the index by 20.65% from 201.4 before any treatment to 159.8 after 90 days of use. Even 30 min after the first application of AFC, most subjects showed an abrupt decrease in the score. This means that the AFC cream has a good effect on the acne erythema and at the same time reduces the skin irritation caused by the anti-acne active ingredients, making it mild for the skin and easy to tolerate.

Various studies have proven the regenerative and soothing effect of each of its components: phytosterol esters from *Crambe abyssinica* [77], *Chlorella vulgaris* extract [78], betaine [79], and vitamin E [80]. It is interesting to note that the erythema value was not increased in any of the test subjects during the AFC application. The lowest decrease was registered after the application of IRC, but no increase in the index was recorded in any of the patients. Only one subject (no. 40—age 32) did not show a change in the erythema value on any of the test days. This proves that IRC is well tolerated even with prolonged use. This is not surprising as its composition is based on non-aggressive ingredients such as niacinamide, panthenol, *Crambe abyssinica* oil, betaine, and rice proteins. Regarding the behaviour of ACC, overall, it reduces the erythema index with the lowest value recorded after the first 10 days of application, a value that is maintained until the end of the study. Nevertheless, 11 (275.5%) of the subjects reported a slight increase in the parameter during the use of ACC, but no visible irritation occurred on the skin surface.

#### 2.9.5. Transepidermal Water Loss (TEWL)

**Figure 13:**
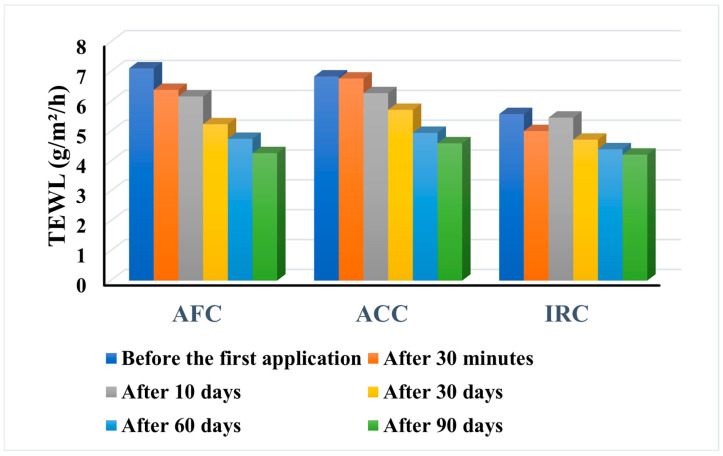
Transepidermal water loss (TEWL).

As a vital component of the body’s metabolism, water is continuously evaporating from the skin. The unit of measurement for water content (Transepidermal Water Loss, or TEWL) is g/m^2^/h. However, water loss will increase as soon as the skin’s barrier function is compromised, even if the damage is minimal and imperceptible to the naked eye. Its measurement is a fundamental measurement in all types of topical product applications and an essential parameter for assessing the skin’s water barrier performance. Skin’s barrier capability and resistance are directly connected, and both are dependent on the stratum corneum’s level of moisture [81]. The results of transepidermal water loss (TEWL) are presented in Figure 13.

The three dermatocosmetic products reduced the TEWL from the initial value to a constant application over 90 days. For all three samples, TEWL decreased even immediately after the first application, and for ACC and AFC, this trend persists, gradually decreasing over time. In the case of IRC, the value recorded after 10 days is higher than the value recorded 30 min after the first application, but from this point onwards it decreases steadily over time. However, some different behaviors were observed. In the case of AFC, subjects no. 1 (age 38) and no. 15 (age 50) showed an increase in TEWL in the first 30 days, and the parameter only began to decrease after 60 days of use. In contrast, subject no. 31 (age 25) showed only a slight change in the indicator, from 4.43 at the beginning of use to 4.37 after 90 days. For ACC, seven of the subjects (175.5%) showed a consistent decrease in TEWL from the first application to the end of the study, while subject no. 37 (age 48) was the only subject to show an increase in the parameter after 90 days of use (from 6.55 to 7.06). For IRC, subjects no. 10 (age 33) and no. 29 (age 45) showed a significant decrease in TEWL from 5.78 and 5.42 at baseline to 3.05 and 3.20, respectively, after three months of daily use.

The results prove a drastic reduction in TEWL after the application of all tested products, which helps to retain hydration and a healthy skin state by forming a protective layer on top of the skin. The skin’s capacity to retain moisture or the integrity of the skin barrier function can be determined by measuring TEWL [82]. Clinical data indicates a strong correlation between acne and skin barrier dysfunction. The skin’s capacity to hold onto moisture is improved by a decrease in TEWL or an enhancement in skin barrier function [83]. Using moisturizers concurrently as an adjuvant acne treatment has therapeutic effects and improves skin condition by reducing inflammation and irritation [84]. As a result, acne and skin conditions can recover rather quickly. The study by Gala M. proved that regular application of moisturizers greatly enhanced skin hydration; repaired or strengthened the skin barrier; decreased skin oiliness; lessened the visibility of acne and prevented the emergence of new acne; decreased skin redness; lessened the visibility of blemishes or lightening them; and lowered the quantity, intensity and visibility of pores.

The registered results are in line with the results of the study by Morin et al., which proved that skin hydration is based on two processes and can thus be explained. The first hydration process takes place in the first few minutes after application of the product and follows first-order kinetics. Their results confirm that the process is primarily related to the filling of cavities and not to hydration-induced changes in the molecular components of the stratum corneum, which would require longer equilibration times. The second hydration process occurs at least one hour after application and has a slower rate with a linear progression. This process is thought to be caused by hydration-related changes in the stratum corneum, including changes in the kinetics and molecular structure of the proteins and lipids of the stratum corneum, the enlargement of corneocytes, and/or the development of water inclusions [85].

#### 2.9.6. Skin Topography

The skin’s surface topography is made up of scales, wrinkles, and lines. A network-like structure made up of primary and secondary lines can be recognized as a polygon. Measurements of skin surface roughness are often used in dermatological treatment and research [86]. The complex structure of human skin, its mechanical behavior, and its microscale topography all play a role in its ability to tolerate topical products. Some dermatological products can damage the skin and impair its functionality [87]. Skin topography analysis is an important test that provides important information about the behavior of the product on the skin during its continuous use.

SELS parameters, surface, texture, and ageing parameters were determined. The SELS parameters (Surface Evaluation of the Living Skin): SEr, SEsc, SEsm, and SEw represent the roughness, desquamation dryness, smoothness, and wrinkles of the skin. A higher SEr value indicates greater roughness, while a lower SEsc value signifies less desquamation/dryness. Conversely, a lower SEsm value indicates smoother skin, and a higher SEw value suggests more wrinkles. The surface parameters are represented by the surface area (if it is high, the skin quality is poor) and the volume (if it is high, the skin is hydrated). The texture parameters are represented by contrast (a low value indicates a good skin condition), entropy (a high value means a well hydrated skin), variance (a high value indicates a rough skin), energy (a high value reveals a young, hydrated, elastic skin) and homogeneity (a high value shows a hydrated skin). The ageing parameters are made up of the anisotropy index, which indicates the directionality of the lines (high values are characteristic of old skin), and the total number of cells—polygons between the lines (a high value is an indicator of young skin).

Black and white images of the skin show wrinkles and black lines, skin peeling represented by very large pixels, and light-coloured areas representing plateaus of the skin’s microrelief. 3D color images show differences in the height and roughness of the skin surface. The results of skin topography parameters for AFC are presented in Table 5.

**Figure 14:**
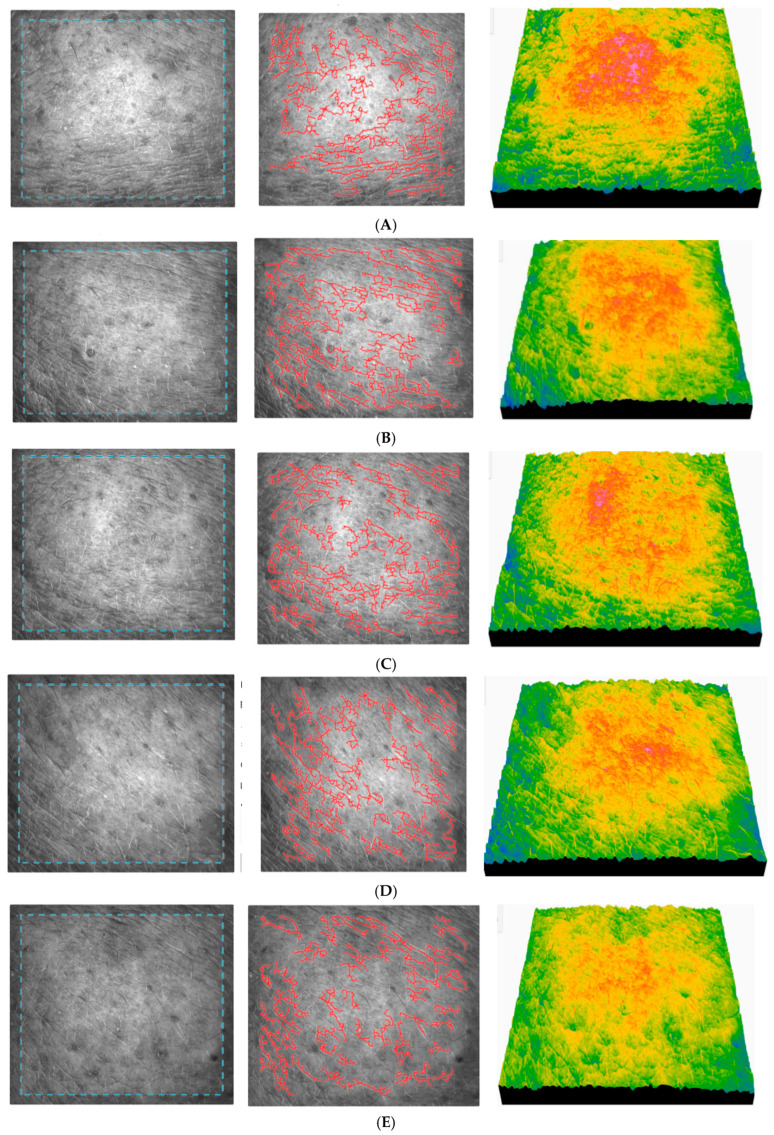

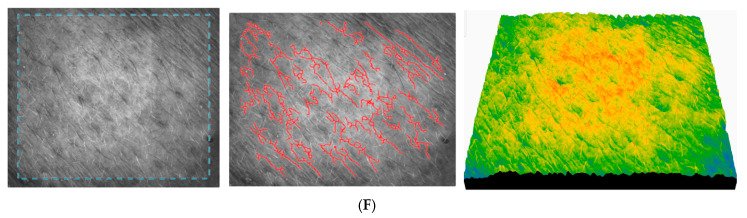
The images registered for subject no. 2 (age 25) when using AFC: (**A**) before the first application; (**B**) at 30 min after the first application; (**C**) after 10 days; (**D**) after 30 days; (**E**) after 60 days; (**F**) after 90 days.

The results of the study show that AFC has a positive effect on the properties of the skin after the first application, which increases with long-term use, resulting in a significant reduction in roughness and dryness and a marked improvement in skin hydration, quality, and suppleness. In addition, it showed a significant anti-ageing effect, which is reflected in the reduction in the SEw value and the anisotropy index, as well as in the increase in the total number of polygons. Figure 14 registered for subject no. 2 (age 25) shows the images of skin topography registered for some subjects during the application of AFC.

The skin before the application of AFC registered for subject no. 2 (age 25) (Figure 14A) shows a moderate roughness, a slightly irregular relief, a slight peeling, a moderately hydrated and smooth skin, and a clear presence of fine wrinkles. It can be seen that skin roughness decreases in the long term, which is a clear sign of improved skin texture. The SEsc parameter gradually decreases and reaches its minimum after 90 days (Figure 14F), which indicates a clear smoothing of the skin. The SEw parameter decreases significantly up to 90 days, indicating a reduction in fine lines. The skin becomes smoother after application of the cream, especially between 30 and 90 days. The texture parameters improve significantly up to 30–90 days, indicating a smoother, more even, and younger texture. The relatively constant number of cells indicates stable epidermal activity, with no signs of inflammation or excessive regeneration.

The 50-year-old subject exhibited skin with mild roughness, indicating moderate skin relief and smoothness, with a moderate to high SEw score indicating the onset of structural changes in the skin (Figure S1 from the Supplementary Materials). The beginnings of photo-ageing are evident, but no significant peeling is observed. After application of the cream, a decrease in skin roughness is observed, and peeling is significantly reduced, indicating good skin hydration with surface smoothing and active cell regeneration. A homogenization of the skin texture and a reduction in the depth of fine lines are also observed, indicating improved hydration, probably also due to the stimulation of collagen synthesis, given the increase in volume.

At baseline, the facial skin of subject 18 (Figure S2 from the Supplementary Materials) has moderate SEr and SEw values, and the skin appears to be fairly smooth and slightly wrinkled. SEsc is very low, which is a good indicator that the skin is well hydrated (no visible scales). The texture appears quite even (moderate contrast and entropy). After applying the cream, the skin condition changes for the better. The surface parameters indicate a smoothing of the macrorelief of the skin and a stabilization of the skin. The texture becomes more homogeneous, smoother, and even, with active skin regeneration and subsequent stabilization. As a conclusion, the skin of all subjects notably improved its microrelief and mechanical properties after the continuous application of AFC during 90 days.

The mechanical properties change in parallel with the biological changes that take place in the epidermis during cell development. The uppermost skin layer and the stratum corneum are commonly identified with different mechanical responses [88]. Compared to the initial physiological state [89], the apparent stiffness of the stratum corneum decreases by orders of magnitude when the corneocytes are hydrated and swell due to water uptake [90]. Due to the presence of essential amino acids (including proline, lysine, and glycine) and peptides in the composition of *Chlorella vulgaris* extract, it has been shown to enhance the production of collagen, increase skin suppleness, and reduce the visibility of wrinkles and fine lines [91]. *Chlorella vulgaris* extract also contains large amounts of polysaccharides that bind moisture, improve the skin barrier, and increase hydration [92]. The vitamin B complex (B1, B2, B3, B6, B12) it contains improves the overall tone and texture of the skin, stimulates cell metabolism, and promotes skin regeneration. Alpha-linolenic acid (ALA), an essential fatty acid, helps to maintain the lipid barrier of the skin, reducing irritation and dryness [93].

The results of skin topography parameters for ACC are presented in Table 6.

**Figure 15:**
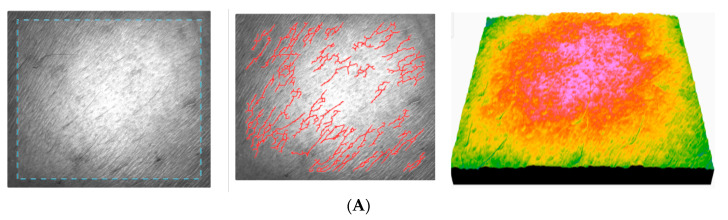

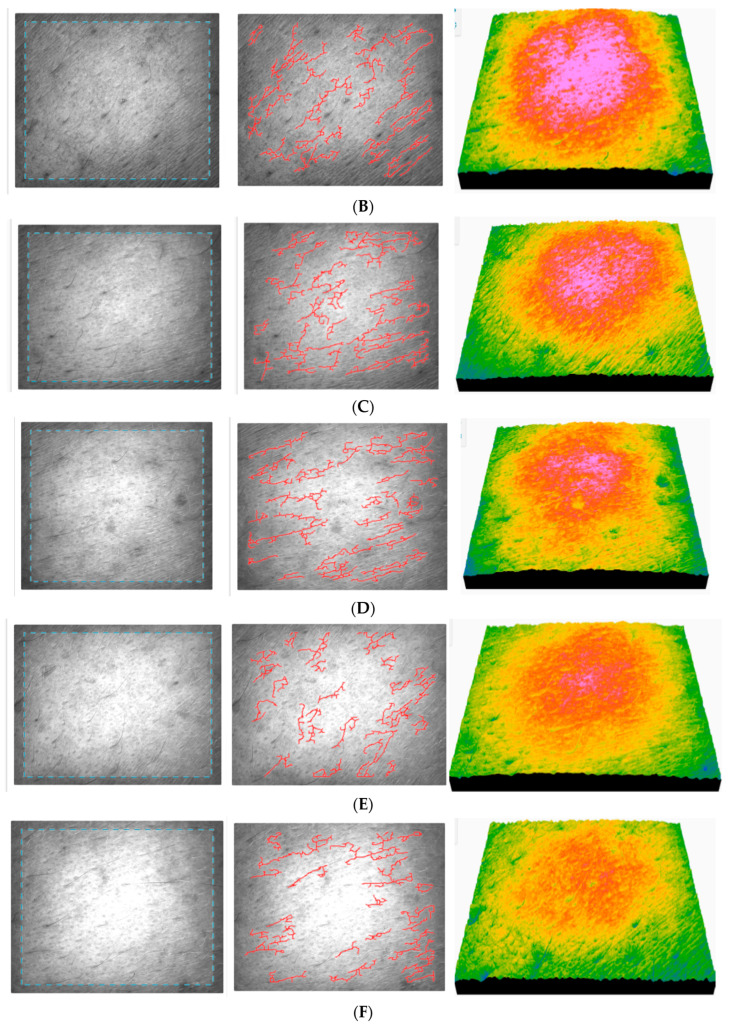
The images registered for subject no. 7 (age 26) when using ACC: (**A**) before the first application; (**B**) at 30 min after the first application; (**C**) after 10 days; (**D**) after 30 days; (**E**) after 60 days; (**F**) after 90 days. Image size: approximately 10 mm × 8 mm.

Similar to AFC performance, ACC also improves the skin’s microrelief and mechanical properties when applied over a longer period of time, but its efficiency is lower. However, evaluation of the skin by light scattering shows a significant decrease in roughness and an increase in smoothness, indicating a more uniform appearance in depth, and the fine lines become less pronounced. The reduction in surface area indicates that the skin becomes more uniform on the surface. The texture parameters indicate that the skin is more moisturized, and the homogeneity of the skin is improved. The change in the total cell count indicates stable cell activity, with a clear improvement, but no greater enhancement than for AFC. Some representative images were selected for the presentation (Figure 15).

With test subject no. 7, both roughness and dryness decrease, the skin becomes smoother, and wrinkles are reduced. The uniformity of the skin also improves, and the relief becomes less pronounced. The texture parameters indicate a natural, more even, and less rigid texture. The total number of cells has increased after a continuous 90-day application of ACC.

In subject no. 26 (Figure S3 from the Supplementary Materials), the behavior is different, and after the application of ACC, an increase in roughness, a strong deterioration of the superficial skin layer towards the end, a possible excessive peeling (dryness), and a decrease in skin smoothness were observed. This indicates a more uneven appearance in depth, and the fine lines become more pronounced, which is a sign of dehydration. The increase in surface area indicates that the skin is becoming more irregular on the surface, and the volume variations emphasize an increase in skin relief. In the texture parameters, it can be observed that the skin becomes more contrasted and “textured”, which is a sign of irregularity, and that the uniformity of texture and homogeneity of the skin decrease. The fluctuations in the total cell count indicate unstable cell activity, with no clear improvement, but no clear regression either. ACC appears to cause irritation, dryness, or aggression of the skin in subject no. 26, aged 49 years. Compared to the other test subjects, however, this appears to be an isolated case, and the behavior could be triggered by a sensitivity to one of the product’s ingredients.

For subject no. 37 (Figure S4 from the Supplementary Materials), the roughness decreases after the constant application of ACC, which means a regeneration of the epidermis and an improvement of the surface. The skin becomes smoother, finer, and the anti-wrinkle effect appears. A temporary inflammatory reaction or structural expansion is observed, but the increase in volume is a sign of hydration and replenishment of the skin. The skin texture parameters indicate a possible unevenness of the texture in certain phases, a temporary unevenness, and a slightly disorganized structure in the long term. The increase in the number of cells is a sign of increased cellular activity and regeneration of the epidermis. The high anisotropy at the end (90 days) could indicate an uneven reorganization of the dermal fibers. Overall, ACC seems to have more positive effects on young people than on older people.

The results of the study by Zhang et al. [94] indicate that rice protein hydrolysate is essential for skin protection due to its high antioxidant capacity, its effective protection of skin tissue, and its inhibition of water loss. Furthermore, the rice protein hydrolysate has been shown to diminish human face wrinkles by 11.8% [95], block the development of aging symptoms in mice [96], and lower the melanin content of human epidermal melanocyte cells [97].

The results of skin topography parameters for IRC are presented in Table 7.

**Figure 16:**
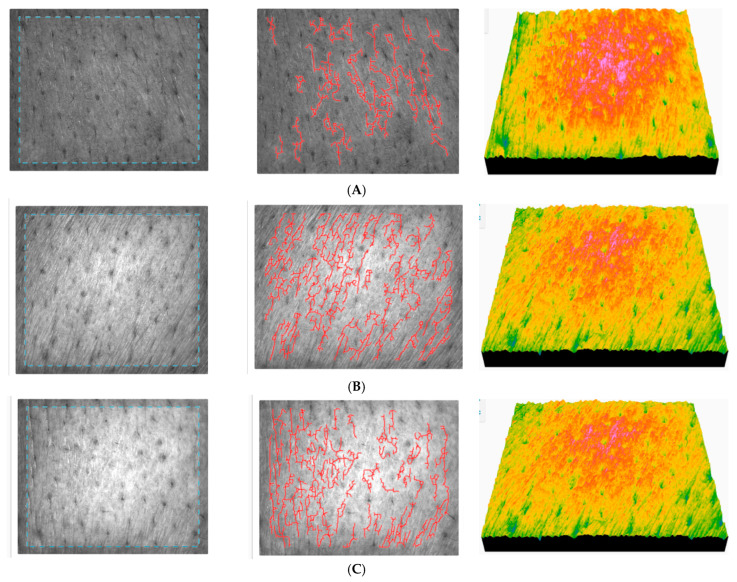

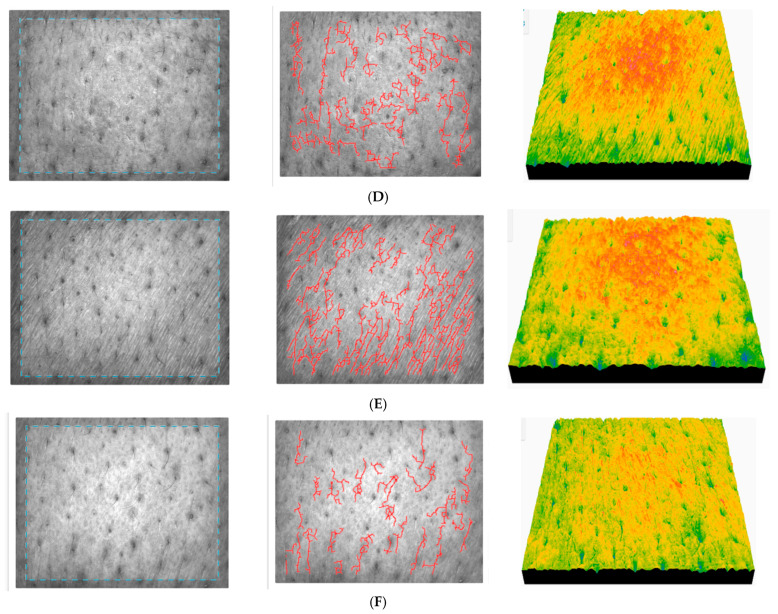
The images registered for subject no. 9 (age 24) when using IRC: (**A**) before the first application; (**B**) at 30 min after the first application; (**C**) after 10 days; (**D**) after 30 days; (**E**) after 60 days; (**F**) after 90 days. Image size: approximately 10 mm × 8 mm.

It can be seen that IRC produces the greatest improvement in skin topography of all three products analyzed. The positive effect was observed from the first application and significantly improved the biophysical properties of the skin during continuous application, with the highest point being reached after 90 days of use. It appears to be suitable for all skin types, both young and mature, with a marked improvement in the overall quality of the skin, especially texture, internal organization, and cellular activity. Roughness slowly diminished and smoothness improved significantly, with fine lines softened in the long term. The internal organization of the skin has improved according to the anisotropy index, and cell activity has considerably improved. The images registered for subject 9 (Figure 16) highlight a positive course with an important reduction in skin roughness. The smoothness of the skin is improved in the long-term application, accompanied by a clear reduction in fine lines and wrinkles. The surface parameters signal a clear improvement in skin uniformity in the long term, and a significant cellular recovery was detected.

The data for subject no. 14 (age 47) (Figure S5 from the Supplementary Materials) indicate that the roughness of the skin has decreased, accompanied by strong hydration, followed by excellent soothing and restoration of smoothness. The fine lines were initially accentuated (signs of dehydration/contracture), but after daily use, they are significantly reduced. Texture is more uniform, balanced, and normalized compared to baseline. The anisotropy index indicates that the internal organization of the skin is largely restored, and the cellular regeneration is active.

The results recorded for subject no. 15 (Figure S6 from the Supplementary Materials) shows a constant improvement in skin quality. The skin is less rough and becomes smoother, while the fine wrinkles are significantly improved. The uniformity of the skin is partially restored, as is hydration, which is also confirmed by the texture parameters. The change in the total cell count attests to clearly activated cell regeneration, a sign of a positive adaptive reaction of the skin.

To summarize, IRC proved to be the most effective of the three products in terms of improving the skin’s microrelief. It was followed by the AFC and then the ACC. The IRC behavior can be explained by the high content of rice protein hydrolysate and vitamin E. Mango butter has also been proven to reduce wrinkles and roughness due to its bioactive components such as phytosterols, tocopherols, and triterpenes [98]. The study conducted by Mandawgade et al. [99] showed that mango butter results in exceptional softness, actively restoring moisture for improved skin protection and rebuilding a naturally occlusive, protective skin barrier, leaving skin feeling smooth, moisturized, and silky. However, Camargo et al. [100] demonstrated that panthenol in skin care formulations has an enhanced protective impact in maintaining skin integrity after 30 days of treatment and leads to a remarkable reduction in TEWL. Also, niacinamide, included in the IRC formulation, was revealed to considerably lower pigmentation, inflammation, and oxidative stress in the skin [101]. Marques et al. stated that niacinamide may be able to partially prevent and/or reverse a number of biophysical alterations linked to skin aging through a variety of multimodal methods [102]. In conclusion, IRC has a very balanced formulation that contributes to strong skin activity by restoring skin functionality and preventing premature ageing.

## 3. Conclusions

The present study shows a comprehensive analysis of three commercially available dermocosmetic gel-cream formulations designed for sensitive and acne-prone skin: ACC (Acne Control Cleanser), AFC (Acne Face Cream), and IRC (gentle cream cleanser serum control, regenerating, hydrating, calming). Through a combined physicochemical assessment, such as spectroscopic analysis (FTIR-ATR), differential scanning calorimetry (DSC), rheological evaluation, spreadability testing, oxidative stability measurements, and antioxidant capacity, the key performance parameters and formulation characteristics that influence their efficacy were evaluated.

The results highlighted that all three gel-cream formulations exhibit pseudoplastic, shear-thinning behaviour, which is advantageous for topical application and ensures ease of spreading. Notably, AFC demonstrated superior oxidative stability, as evidenced by its extended induction period, and maintained a robust antioxidant capacity. In contrast, although IRC provided excellent spreadability and cosmetic efficacy, it was ideal for extensive skin coverage and prolonged hydration. ACC, with its formulation rich in vegetable oils and active constituents such as rice proteins and vitamin E, offers rapid absorption.

These results emphasise the importance and synergy of formulation composition and used ingredients, by influencing the overall performance of topical cosmetic products. The differences observed among ACC, AFC, and IRC highlight that not only the presence but also the compatibility and solubility of active ingredients within the formulation matrix are essential for achieving the desired use and sensory properties. In conclusion, the present study contributes to the optimization of dermocosmetic formulations. The in vivo investigations of the cosmetic products’ behaviour demonstrate a good efficiency on the skin as they were designed.

## 4. Materials and Methods

### 4.1. Materials

Three dermatologically tested topical gel-cream formulations developed by Brand Chanand^®^: Acne Control Cleanser (ACC), Acne Face Cream (AFC), and Gentle Cream Cleanser Serum Control, Regenerating, Hydrating, Calming (IRC), were used in the present study. All products are recommended for sensitive skin and are formulated with synergistic blends of bioactive compounds designed for cleansing, moisturizing, and therapeutic purposes [103].

### 4.2. Methods

#### 4.2.1. Physico-Chemical Determinations

##### Organoleptic Characteristics

Physico-chemical testing of the gel-cream formulations was performed to evaluate their physical properties such as color, odor, and texture [10].

##### Determination of Density and pH

The pH was measured using a calibrated ExStik pH meter (model PH110) (Extech, Taiwan, Taiwan), under controlled laboratory conditions. Density was determined by applying the classical formula, defined as the ratio of mass to volume, using precisely weighed and volumetrically measured cream samples. Each determination was made for three experiments, and the results are expressed as mean ± standard deviation.

##### Spreadability Study

Spreadability was assessed using the extensometric method with the Ojeda Arbussa apparatus, which quantifies the spreading area under standardized mechanical pressure, at five points. On a glass plate, 1 g of each hydrogel was placed in the middle of a circle with a diameter of 2 cm. After a second glass plate with a 182.35 g weight was applied to the surface, the diameter of the circle filled by the gel was measured. After a 2-min rest period, weights weighing 50 g, 100 g, 150 g, 200 g, and 500 g were placed successively on the upper glass plate, and measurements were taken after each individual weight was added. The diameter of the circle occupied by the gel was then measured [104]. The following formula (Equation (1)) was used to determine the spreading area occupied by the hydrogel:

(1) $$S=\pi r^{2}$$

where *S* is the spreading area (mm^2^) and *r* is the radius (mm).

##### Oxidative Stability Test

Lipid auto-oxidation is an important factor that can significantly reduce the shelf life and efficacy of cosmetic products, especially formulations rich in unsaturated oils or active ingredients sensitive to oxygen. To evaluate the oxidative stability, expressed as induction period, of the studied gel-cream formulations, the Velp OXITEST reactor (Velp Scientifica, Usmate, Italy), which accelerates the oxidation process under controlled and reproducible conditions, was used. The test involves subjecting the cream samples to elevated oxidative stress by exposing them to a temperature of 90 °C and a pressure of 6 atm in the presence of pure oxygen. Small amounts of product (5–10 g) were placed in the oxidation chamber, where oxygen consumption is continuously monitored. As the oxidation reaction progresses, oxygen is consumed, leading to a decrease in internal pressure. The sharp drop in oxygen pressure indicating the onset of significant oxidation was determined using the OXISoft™ 3.0.0 software, which is integrated with the Velp OXITEST system.

The induction period (IP) is determined as the time (in hours and minutes) until a sharp drop in oxygen pressure is detected, signalling the onset of significant oxidation. This point correlates with the formation of primary oxidation products, such as hydroperoxides, and is commonly associated with early signs of rancidity or changes in sensory and functional properties. Two calculation methods were considered for determining the IP (i), the least squares method, which fits a curve to the oxygen consumption data, and (ii) the graphical method used as a supplementary recalculation approach if needed.

A longer induction period indicates a higher resistance of the gel-cream to oxidative degradation, and thus, greater shelf-life stability under real storage conditions. This parameter is particularly relevant for formulations containing plant oils, unsaturated fatty acids, or antioxidants, and is essential for determining packaging, storage, and formulation optimization.

##### Fourier Transform Infrared Spectroscopy (FTIR-ATR) Analysis

Fourier-transform infrared (FTIR) spectra were recorded using a JASCO FT/IR 4700 spectrophotometer (Tokyo, Japan) equipped with a monolithic diamond attenuated total reflectance (ATR) accessory (Tokyo, Japan). Spectra were collected over the wavenumber range of 4000–400 cm^−1^ at a 45° incident angle, with 64 scans accumulated at a resolution of 4 cm^−1^.

##### Differential Scanning Calorimetry (DSC) Analysis

Differential scanning calorimetry (DSC) analyses were carried out using a Mettler Toledo DSC 3 calorimeter (Greifensee, Switzerland), under 100 mL min^−1^ nitrogen flow, at a heating rate of 10 °C min^−1^. Optical microscopy images were acquired in situ, during the DSC analysis, using an Olympus SC50 digital microscope (Tokyo, Japan), at a frame rate of 1 image min^−1^.

##### Rheology

Rheological measurements were performed using a B-One Plus rotational viscometer (Lamy Rheology Instruments, Champagne du Mont d’Or, France) equipped with a TE 95 spindle. The specified amount of cream was introduced into the measurement vessel, and the spindle was immersed up to the marked level to ensure reproducibility and accurate contact. Tests were conducted at various rotational speeds: 50, 100, 150, 200, and 250 rpm, with each speed maintained for 20 s. The temperature was kept constant at 22 °C throughout the experiment to eliminate viscosity variations due to thermal fluctuations. The hysteresis curves, represented as the variation in shear stress as a function of shear rate during ascending and descending shear cycles, were evaluated to determine the rheological properties of the analysed samples.

#### 4.2.2. Antioxidant Activity

Three methods (ABTS, DPPH, and FRAP) were used to evaluate the antioxidant activity of the cosmetic products, and the results were compared with ascorbic acid used as a positive control. DPPH, ABTS, and TPTZ were purchased from Sigma-Aldrich Chemie GmbH, Schnelldorf, Bayern, Germany.

##### Preparation of the Samples

1 g of each product was mixed with 10 mL of ethanol and sonicated for 15 min, then centrifuged at 7500 rpm for 15 min. Finally, the mixtures were filtered through a 0.45 μm cellulose acetate filter.

##### DPPH Radical Scavenging Activity

It was determined according to the method established by Brand-Williams et al. [105]. The sample solutions were mixed with DPPH solution and sonicated for 10 min. At 517 nm, the absorbance values were measured every five minutes for 30 min, and the results are expressed as the percentage decrease in optical density from the starting value.

The DPPH scavenging efficiency (radical scavenging activity (RSA%) was calculated according to Equation (2):

(2) $$RSA\%=\frac{\left(Abs_{0}-Abs_{p}\right)}{Abs_{0}}\times 100$$

where *Abs*_0_ is the absorbance of DPPH solution (reference) at *t*_0_ (initial optical density); *Abs*_p_ is the absorbance of the samples measured at different periods of time *t* (sample optical density).

##### ABTS Radical Scavenging Activity

A previously described method was used to evaluate the total antioxidant capacity [106]. ABTS and potassium persulfate reacted for 12 h at room temperature in the dark to form the cationic radical ABTS^●+^. At 734 nm, the absorbance of the working reagent was 0.70 ± 0.02. After the addition of the samples, the decrease in absorbance at 734 nm was measured after 5 and 10 min, and the results were calculated in relation to a Trolox calibration curve. The final data were displayed as mmol Trolox equivalents/mg product.

##### FRAP Assay

The FRAP assay was evaluated according to the method described by Benzie IFF and Strain JJ [107]. 5 and 30 min after preparation, the absorbances at 593 nm were measured for the samples combined with FRAP solution. Fe^2+^ calibration curves in methanol were used to calculate the FRAP values (µmol equivalent of Fe^2+^ (FeSO_4_)/L).

All analyses were performed in triplicate. The results are presented as mean values with corresponding error bars representing standard deviations.

#### 4.2.3. In Vivo Investigations of the Cosmetic Products’ Behavior

Various biophysical properties of the skin were determined with the appropriate Multi Probe Adapter MPA 6 device (manufactured by Courage + Khazaka electronic GmbH, Cologne, Germany) probes: pH (with Skin-pH-Meter PH 905 (Cologne, Germany)), hydration level (with Corneometer^®^ CM 825), melanin content and degree of erythema (with Mexameter^®^ MX 18, Courage + Khazaka electronic GmbH, Cologne, Germany) and transepidermal water loss (with Tewameter^®^ TM Hex, Courage + Khazaka electronic GmbH, Cologne, Germany). Skin topography was determined by image analysis with the Visioscan^®^ VC 20plus, also manufactured by Courage + Khazaka electronic GmbH, Cologne, Germany.

##### Study Design

The experiments were carried out on the facial skin of 40 healthy volunteers. The test subjects, who identified themselves as women, were between 18 and 50 years old, with a mean age of 35.25 ± 17.25. They were informed about the products to be applied to the skin and gave their informed verbal and written consent. Each volunteer was given a code number (1–40) according to their enrollment order. The subjects declared that they did not suffer from any skin disease or sensitivity. Eight of the volunteers were smokers, and two of them were vegetarians.

The analyses were performed at the same time (9 am), under the same temperature (22 ± 0.5 °C) and humidity conditions (45%), on the forehand, right and left cheeks, at a distance of at least 5 cm from the eyes. The subjects applied the products every evening before going to bed and left them on the skin surface all night (around 7 h). During this time, they were asked not to use any other skin products. The following test areas were considered: AFC was applied on the right cheek, AFC on the left cheek, and IRC on the forehand. The application area was 4 × 4 cm^2^, which ensured sufficient clearance for the measurement probe inside each site (the probes are approximately 12 cm in diameter). The measurements were realized at six different times: before the first application, after 30 min, 10 days, 30 days, 60 days, and 90 days after the first application. The results are given as mean ± SD.

The study was conducted in accordance with the Declaration of Helsinki and approved by the Commission for University and Scientific Ethics and Deontology of the University of Medicine and Pharmacy of Craiova (no. 72/11 May 2021).

## Figures and Tables

**Table 1:** The organoleptic evaluation of gel-cream formulations.

Products	Color	Odor	Texture
ACC	white	odor characteristic of the components	Soft, greasy, and relatively easily absorbed into the skin
AFC	white	odor characteristic of the components	More compact, less greasy, and relatively easily absorbed into the skin
IRC	white-gray	odor characteristic of the components	Slightly oily, fluid

**Table 2:** The density and pH values of gel-cream formulations.

Products	Density (g/cm^3^) *	pH (22 °C) *
ACC	0.62 ± 0.02	6.02 ± 0.03
AFC	0.99 ± 0.03	5.81 ± 0.02
IRC	0.73 ± 0.01	5.05 ± 0.02

* Data are expressed as mean ± SD (*n* = 3).

**Table 3:** Induction period values of the studied gel-cream formulations.

Products	Induction Period (min)
ACC	28.54
AFC	64.38
IRC	0.40

**Table 4:** Thermal data and water content are computed from the water heat of fusion values.

Products	H_2_O Melting		Organic Melting		H_2_O Evaporation		H_2_O (%wt.)
	*T* (°C)	Δ*H* (Jg^−1^)	*T* (°C)	Δ*H* (Jg^−1^)	*T* (°C)	Δ*H* (Jg^−1^)	
ACC	−2.9	136.4	29.8	103.3	100.1	400.2	40.8
AFC	−2.8	194.1	39.1	1290.7			58.1
IRC	−6.3	85.0	40.4	60.2	103.1	316.0	25.5

**Table 5:** Skin topography indicators for AFC.

Parameter		Before the First Application	After 30 min	After 10 Days	After 30 Days	After 60 Days	After 90 Days
SELS	SEr	1.76 ± 0.91	2.38 ± 1.11	1.91 ± 0.71	1.94 ± 0.58	2.02 ± 0.49	2.53 ± 0.83
	SEsc	1.10 ± 0.04	0.06 ± 0.01	0.04 ± 0.03	0.02 ± 0.02	0.01 ± 0.01	0.01 ± 0.01
	SEsm	498.12 ± 21.25	495.21 ± 12.44	424.48 ± 27.19	439.42 ± 19.68	406.27 ± 15.61	385 ± 18.47
	SEw	159.71 ± 15.86	158.8 ± 4.75	121.45 ± 8.18	115.96 ± 10.85	104.55 ± 9.52	82.96 ± 8.81
Surface parameters	Surface area	655.74 ± 37.94	614.28 ± 28.44	620.07 ± 9.57	603.16 ± 21.18	583.66 ± 25.13	554.55 ± 17.40
	Volume	101.07 ± 22.46	111.47 ± 16.29	112.89 ± 0.14	117.48 ± 23.79	119.51 ± 18.75	122.01 ± 21.39
Texture parameters	Contrast	1.27 ± 0.22	1.08 ± 0.65	1.19 ± 0.71	1.11 ± 0.33	1.09 ± 0.28	1.05 ± 0.17
	Entropy	1.51 ± 0.57	1.64 ± 0.82	1.55 ± 0.77	1.54 ± 0.85	1.57 ± 0.82	1.61 ± 0.62
	Variance	4.65 ± 1.66	4.19 ± 1.21	4.38 ± 1.35	4.43 ± 1.29	4.55 ±1.85	4.50 ± 1.67
	Energy	0.02 ± 0.01	0.02 ± 0.01	0.02 ± 0.01	0.02 ± 0.01	0.02 ± 0.01	0.02 ± 0.01
	Homogeneity	1.4 ± 0.85	1.37 ± 0.99	1.38 ± 0.87	1.48 ± 0.54	1.47 ± 0.69	1.48 ± 0.71
Anisotropy	Anisotropy index	20.85 ± 8.69	19.42 ± 7.15	18.99 ± 8.48	17.89 ± 5.38	18.22 ± 8.04	17.51 ± 6.29
	The total number of cells (polygons)	59 ± 13	61 ± 18	70 ± 15	72 ± 16	75 ± 13	75 ± 9

**Table 6:** Skin topography indicators for ACC.

Parameter		Before the First Application	After 30 min	After 10 Days	After 30 Days	After 60 Days	After 90 Days
SELS	SEr	1.92 ± 0.83	2.01 ± 1.05	2.05 ± 0.97	2.03 ± 0.67	2.12 ± 0.84	2.15 ± 0.92
	SEsc	0.93 ± 0.52	0.54 ± 0.44	0.53 ± 0.28	0.37 ± 0.20	0.31 ± 0.14	0.21 ± 0.07
	SEsm	514.75 ± 29.18	417.93 ± 34.02	482.96 ± 17.25	450.48 ± 26.94	447.15 ± 13.72	405.81 ± 19.14
	SEw	164.6 ± 10.23	159.14 ± 15.15	147.77 ± 12.45	139.99 ± 20.47	131.12 ± 17.61	128.97 ± 9.44
Surface parameters	Surface area	690.61 ± 41.13	601.14 ± 29.52	648.08 ± 27.11	639.79 ± 34.58	638.10 ± 28.36	626.02 ± 30.02
	Volume	101.16 ± 13.20	103.21 ± 12.78	103.88 ± 17.63	111.70 ± 14.22	112.74 ± 20.96	118.80 ± 18.19
Texture parameters	Contrast	1.35 ± 0.41	0.97 ± 0.68	1.24 ± 0.55	1.16 ± 0.70	1.14 ± 0.59	1.09 ± 0.65
	Entropy	1.44 ± 0.75	1.42 ± 0.49	1.41 ± 0.62	1.39 ± 0.57	1.40 ± 0.68	1.36 ± 0.66
	Variance	5.08 ± 2.01	5.11 ± 1.77	4.95 ± 1.53	4.74 ± 1.08	4.58 ± 1.24	4.45 ± 1.62
	Energy	0.01 ± 0.00	0.01 ± 0.00	0.01 ± 0.00	0.01 ± 0.00	0.01 ± 0.00	0.02 ± 0.01
	Homogeneity	1.39 ± 0.48	1.43 ± 0.27	1.39 ± 0.72	1.39 ± 0.83	1.40 ± 0.76	1.43 ± 0.44
Anisotropy	Anisotropy index	24.47 ± 3.09	21.68 ± 3.74	24.11 ± 5.13	21.94 ± 4.95	20.87 ± 3.86	20.8 ± 4.07
	The total number of cells (polygons)	57 ± 16	67 ± 18	60 ± 17	61 ± 12	64 ± 11	67 ± 17

**Table 7:** Skin topography indicators for IRC.

Parameter		Before the First Application	After 30 min	After 10 Days	After 30 Days	After 60 Days	After 90 Days
SELS	SEr	1.81 ± 0.25	2.10 ± 0.36	2.73 ± 0.54	2.98 ± 0.28	2.85 ± 0.60	4.06 ± 0.52
	SEsc	0.88 ± 0.19	0.64 ± 0.11	0.56 ± 0.20	0.50 ± 0.16	0.44 ± 0.09	0.43 ± 0.11
	SEsm	428.12 ± 27.18	404.95 ± 17.26	378.29 ± 20.15	366.40 ± 14.74	290.48 ± 12.03	251.01 ± 23.55
	SEw	137.97 ± 33.14	107.94 ± 25.32	100.12 ± 19.78	97.94 ± 12.96	96.55 ± 16.75	60.14 ± 21.44
Surface parameters	Surface area	796.17 ± 43.23	755.32 ± 27.42	759.08 ± 37.55	735.15 ± 29.14	684.56 ± 22.68	672.5 ± 20.91
	Volume	112.75 ± 14.48	126.32 ± 16.51	116.61 ± 12.16	116.06 ± 25.39	116.76 ± 23.08	128.21 ± 18.77
Texture parameters	Contrast	2.01 ± 0.30	1.51 ± 0.24	1.99 ± 0.11	1.87 ± 0.33	1.67 ± 0.28	1.10 ± 0.27
	Entropy	1.41 ± 0.17	1.35 ± 0.08	1.39 ± 0.15	1.36 ± 0.04	1.36 ± 0.12	1.37 ± 0.23
	Variance	6.98 ± 0.75	6.69 ± 0.35	6.02 ± 0.61	5.76 ± 0.46	5.61 ± 0.50	4.75 ± 0.27
	Energy	0.01 ± 0.00	0.01 ± 0.00	0.01 ± 0.00	0.02 ± 0.01	0.02 ± 0.01	0.02 ± 0.01
	Homogeneity	1.33 ± 0.18	1.39 ± 0.26	1.39 ± 0.45	1.46 ± 0.38	1.47 ± 0.49	1.52 ± 0.35
Anisotropy	Anisotropy index	26.3 ± 5.04	24.06 ± 3.78	23.12 ± 5.01	22.91 ± 3.98	21.47 ± 3.26	21.28 ± 4.15
	The total number of cells (polygons)	73 ± 13	76 ± 10	82 ± 14	81 ± 15	88 ± 11	90 ± 16

## Data Availability

Data are contained within the manuscript.

## Supplementary Materials

The following supporting information can be downloaded at: https://www.mdpi.com/article/10.3390/gels11070532/s1, Figure S1: The images registered for subject no. 15 (age 50) when using AFC: (**A**) before the first application; (**B**) at 30 min after the first application; (**C**) after 10 days; (**D**) after 30 days; (**E**) after 60 days; (**F**) after 90 days; Figure S2: The images registered for subject no. 18 (age 35) when using AFC: (**A**) before the first application; (**B**) at 30 min after the first application; (**C**) after 10 days; (**D**) after 30 days; (**E**) after 60 days; (**F**) after 90 days; Figure S3: The images registered for subject no.26 (age 49) when using ACC: (**A**) before the first application; (**B**) at 30 min after the first application; (**C**) after 10 days; (**D**) after 30 days; (**E**) after 60 days; (**F**) after 90 days; Figure S4: The images registered for subject no. 37 (age 48) when using ACC: (A) before the first application; (**B**) at 30 min after the first application; (**C**) after 10 days; (**D**) after 30 days; (**E**) after 60 days; (**F**) after 90 days; Figure S5: The images registered for subject no. 15 (age 50) when using IRC: (**A**) before the first application; (**B**) at 30 min after the first application; (**C**) after 10 days; (**D**) after 30 days; (**E**) after 60 days; (**F**) after 90 days; Figure S6: The images registered for subject no. 15 (age 50) when using IRC: (**A**) before the first application; (**B**) at 30 min after the first application; (**C**) after 10 days; (**D**) after 30 days; (**E**) after 60 days; (**F**) after 90 days. Table S1: Composition of gel-cream formulations according to Chanand; Table S2: The functional groups for the ACC nourishing formulation, their peak position, and the identified possible source ingredients; Table S3: The functional groups for the AFC anti-acne cream formulation, the peak position, and possible source ingredients; Table S4: The functional groups for the IRC formulation, their peak position, and the identified possible ingredients.
