# Supplementary material for: Comparative Physicochemical and Pharmacotechnical Evaluation of Three Topical Gel-Cream Formulations
Source: Gels. 2025 Jul 9;11(7):532. doi: 10.3390/gels11070532 (PMC12295134; doi:10.3390/gels11070532)
Supplement: Supplementary file 1 [file gels-11-00532-s001.zip › gels-3714626-supplementary.pdf]

Article

Comparative Physicochemical and Pharmacotechnical Evaluation of Three Topical Gel-Cream Formulations

Supplementary Materials

Table S1. Composition of gel-cream formulations according to Chanand [https://chanand.com].

Products	Composition
ACC	Acne control cleanser a multi-functional facial cleanser developed to gently yet deeply cleanse the skin. It removes dead skin cells, oxidized sebum, pollutants, and makeup, while maintaining the hydration and microbiome balance of the skin. Its active ingredients include rice proteins, which cleanse without causing dehydration; Mango seed butter, known for preventing premature aging and supporting skin regeneration and collagen synthesis; Abyssinian seed oil, which effectively eliminates impurities while nourishing and hydrating the skin; Vitamin E, a potent antioxidant that reduces inflammation and promotes youthful appearance. According to the manufacturer, after one day of twice-daily application, ACC eliminates 100% of oxidized sebum and impurities, while after five days, skin hydration increases by 80%.
AFC	Anti-acne face cream containing four synergistic active ingredients that help balance the skin microbiome, reduce inflammation, and enhance skin radiance. The formulation includes Chlorella vulgaris extract, a green microalga that revitalizes the skin and reduces sebum production and comedones; Phytosterol esters from Crambe Abyssinica, which restore the skin's natural barrier and provide a luminous, hydrated appearance; Betaine, which ensures proper hydration and protects skin cells from dehydration; Vitamin E, which supports collagen integrity and acts as a natural antioxidant. Clinical data provided by the manufacturer indicate that, following a single day of twice-daily use, AFC reduces inflammation linked to microbiome imbalance by 61.3%. After two weeks, skin luminosity increases by 42%, and comedone count is reduced by 16% after 28 days.
IRC	Intensive Repair Complex Gentle Cream Cleanser is a high-end cleanser formulated for all skin types, particularly sensitive skin. It removes dirt, makeup, excess sebum, and pollutants while preserving the skin's natural pH and barrier integrity. The product is enriched with ten active ingredients, including Niacinamide, which enhances antimicrobial peptide release and reduces pore size; Blueberry oil, with soothing and antioxidant properties; Abyssinian seed oil, betaine, and panthenol,

	which together hydrate, calm, and protect sensitive or damaged skin; Squalene, which helps neutralize UV-induced skin damage; Rice protein and mango seed butter, for gentle cleansing and collagen support; Vitamin E, which reduces inflammation and promotes a rejuvenated appearance; a clean, allergen-free fragrance derived from Aloe and Mango. According to clinical data, IRC increases keratinocyte proliferation by 44% and fully restores the skin barrier within 72 hours. After 30 days of continued application, symptoms of eczema and psoriasis are reduced by 42.8%.

Table S2. The functional groups for the ACC nourishing formulation, their peak position, and the identified possible source ingredients.

Wavenumber (cm^{-1})	Functional groups	Ingredients
3291.9 cm^{-1}<br>3226.3 cm^{-1}	broad O–H and<br>N–H stretching vibration	oils, rice protein, and mango seed butter
2914.9 cm^{-1}<br>2849.3 cm^{-1}	C–H stretch (from alkanes)	Fatty acids/oils (Abyssinian oil, mango seed butter)
1729.8 cm^{-1}	C=O stretch (ester or lipid carbonyl)	Mango seed butter
1640 cm^{-1}	Amide I (C=O in proteins)	Rice protein
1469.5 cm^{-1}<br>1392.4 cm^{-1}	CH_2 and CH_3 bending	Lipids and fatty chains from oils
between 1300-1050 cm^{-1}	C–O and C–N stretches	esters

Table S3. The functional groups for the AFC anti-acne cream formulation, the peak position, and possible source ingredients.

Wavenumber (cm^{-1})	Functional groups	Ingredients
3296.7 cm-1	broad O–H<br>N–H stretching vibration (H-bonded)	Betaine, Chlorella extract, Crambe oil esters (hydration/moisture)
2923.6 cm-1<br>2853.2 cm-1	C–H asymmetric stretch (CH2)<br>C–H symmetric stretch (CH3)	Fatty acids, phytosterol esters, and vitamin E, oils/lipids
1743.3 cm-1	C=O stretch (ester or triglycerides)	Phytosterol esters, vitamin E, plant oils
1636.3 cm-1	Amide I (C=O stretch in proteins)	Chlorella extract, vitamin E (aromatic ring)
1457 cm-1	CH2 and CH3 bending	vitamin E, phytosterols

1400 cm-1	COO^- symmetric stretch	Betaine and carboxylate compounds
1338.4 cm-1	C–N or C–H stretches	Betaine, Chlorella extract
1208 cm-1 and 1160 cm-1	C–O stretch (esters)	phytosterol esters
1044.3 cm-1	C–O stretching (alcohols/ethers) or C–N stretches	moisturizers, esters, betaine

Table S4. The functional groups for the IRC formulation, their peak position, and the identified possible ingredients.

**Wavenumber (cm^{-1})**	**Functional groups**	**Ingredients**
3294.8 cm^{-1} and 3222.5 cm^{-1}	broad O–H and N–H stretching vibrations	Niacinamide, betaine, panthenol, mango seed butter, aloe
2915.6 cm^{-1} and 2850.3 cm^{-1}	Long-chain aliphatic hydrocarbon C–H stretch (CH_2 and CH_3 from alkanes)	Squalene, oils, mango seed butter
1729.8 cm^{-1}	C=O stretch (ester and carboxylic acid)	Mango seed butter, panthenol, vitamin E, esters
1636.3 cm^{-1}	Amide I (C=O stretch)	Rice protein, niacinamide
1469.5 cm^{-1}<br>1394.3 cm^{-1}	CH_2 and CH_3 bending deformations	Lipids, betaine, squalene, and rice protein components
1330.5 cm^{-1}	C–N or C–H stretching vibrations	Niacinamide, betaine, protein derivatives
1215.9 cm^{-1} and 1180.2 cm^{-1}	C–O stretch (esters and alcohols)	Panthenol, betaine, esterified plant lipids
1049.1 cm^{-1}	C–O stretching and C–N stretching vibrations	Alcohols, betaine, glycerol

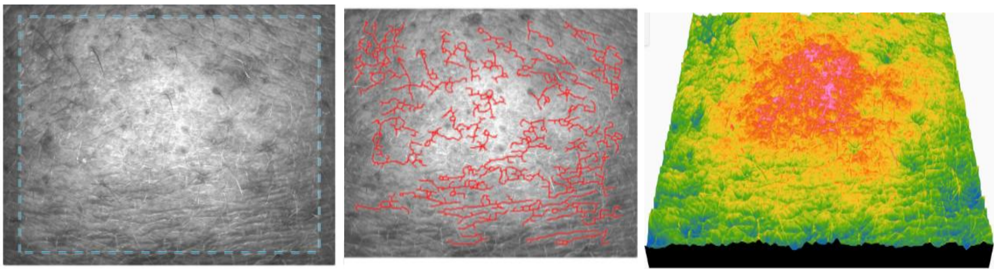

A

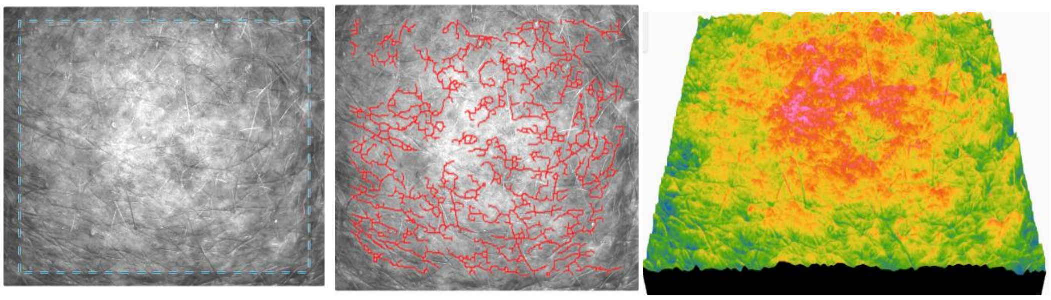

B

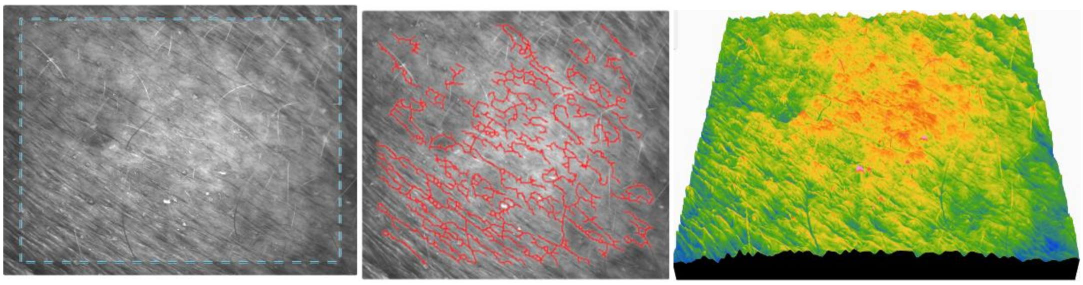

C

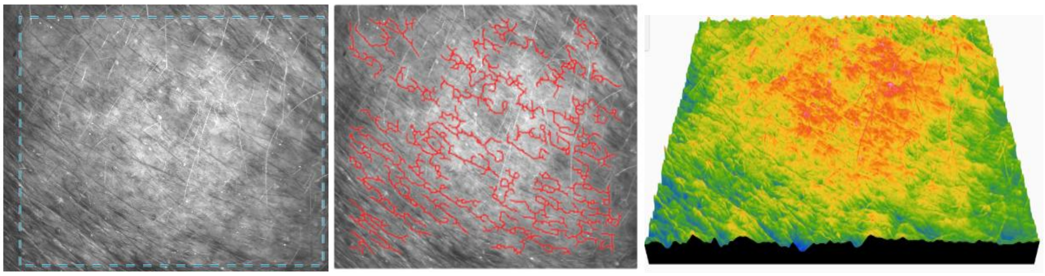

D

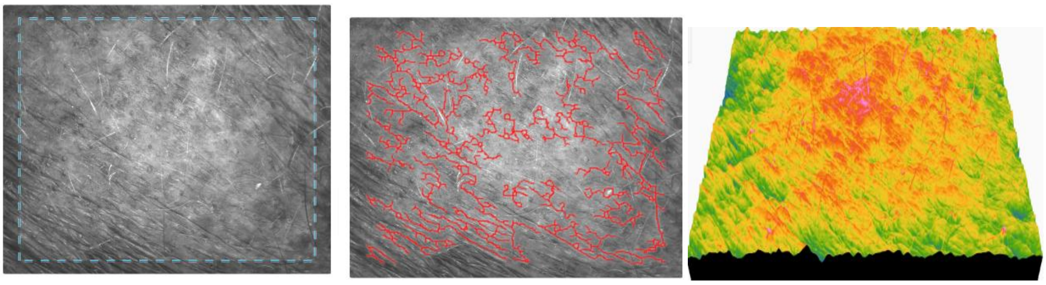

E

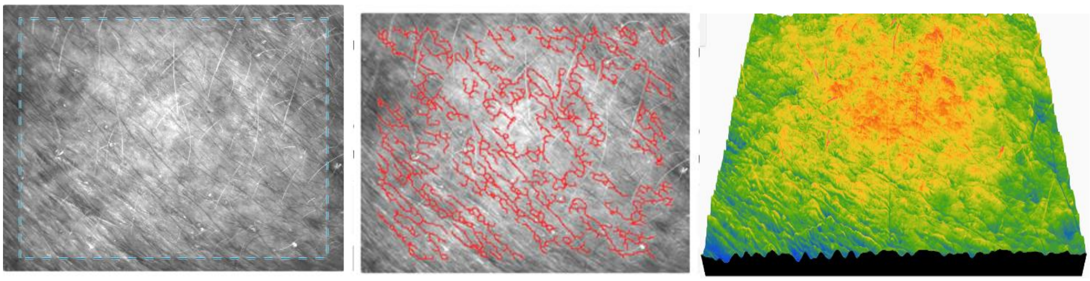

F

Figure S1. The images registered for subject no. 15 (age 50) when using AFC: A. before the first application; B. at 30 minutes after the first application; C. after 10 days; D. after 30 days; E. after 60 days; F. after 90 days.

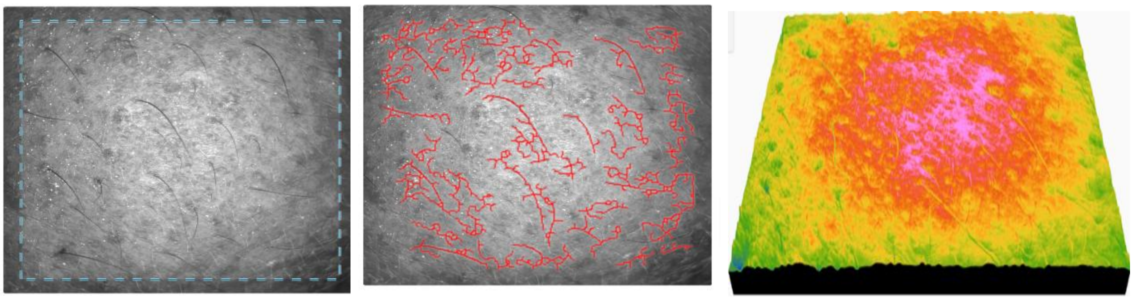

A

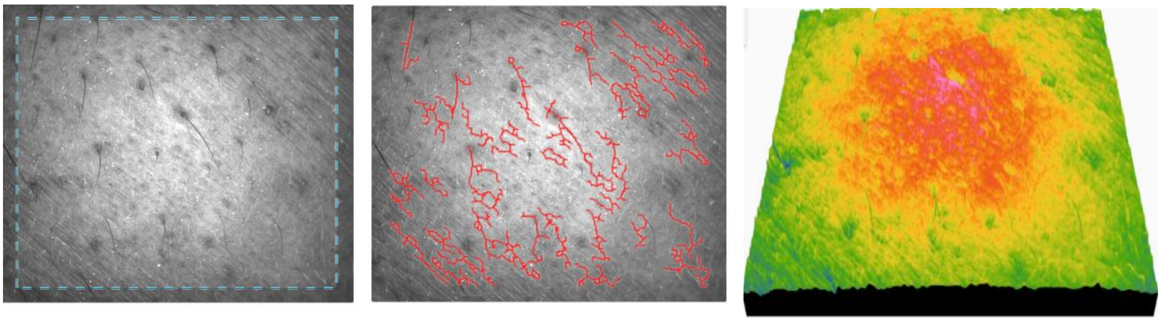

B

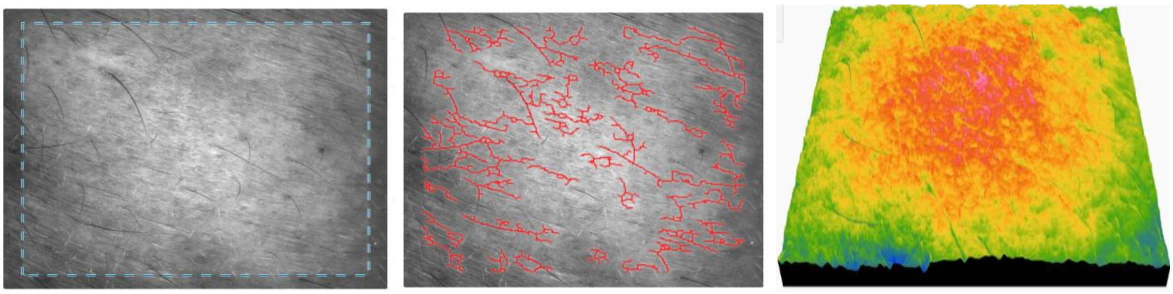

C

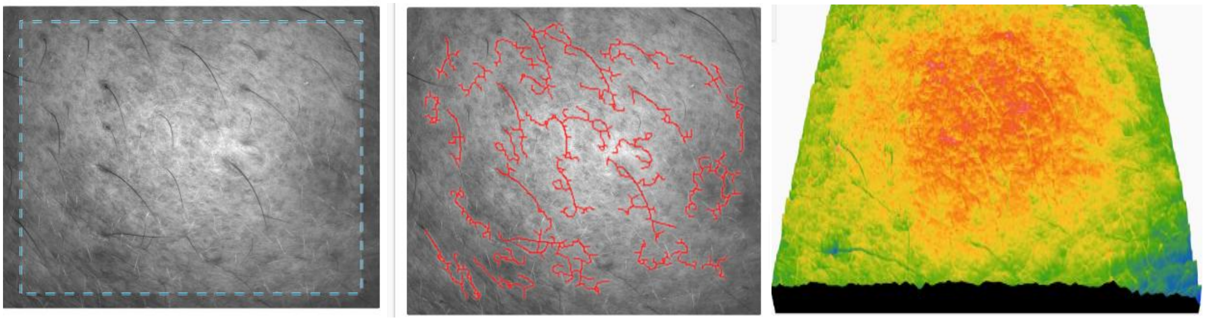

D

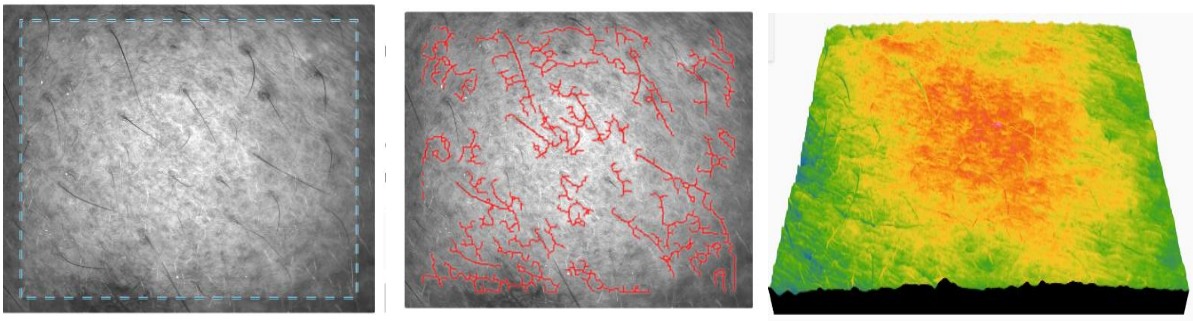

E

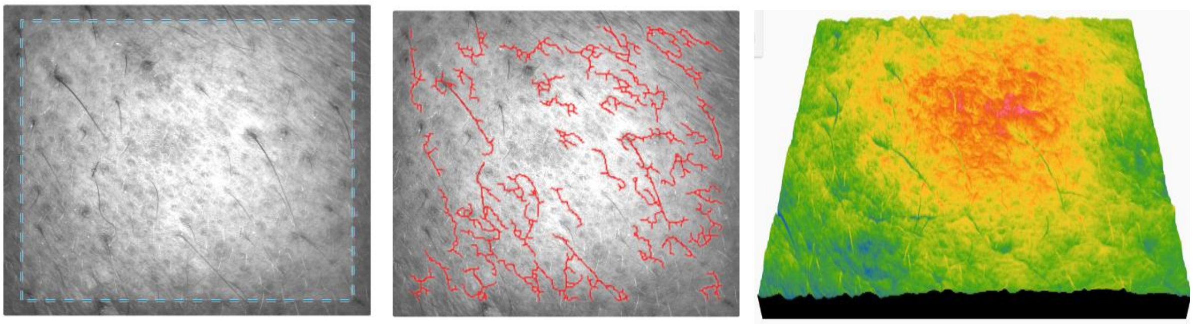

F

Figure S2. The images registered for subject no. 18 (age 35) when using AFC: A. before the first application; B. at 30 minutes after the first application; C. after 10 days; D. after 30 days; E. after 60 days; F. after 90 days.

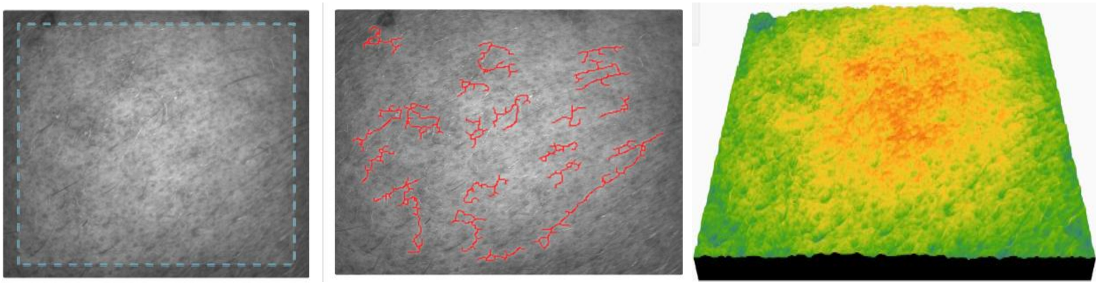

A

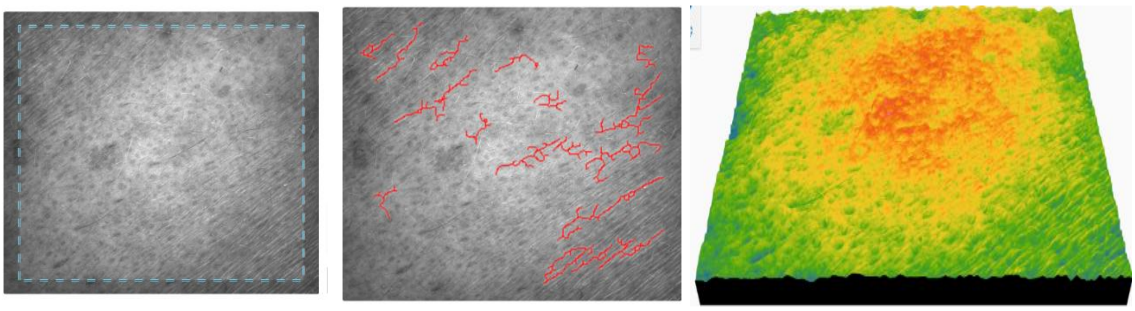

B

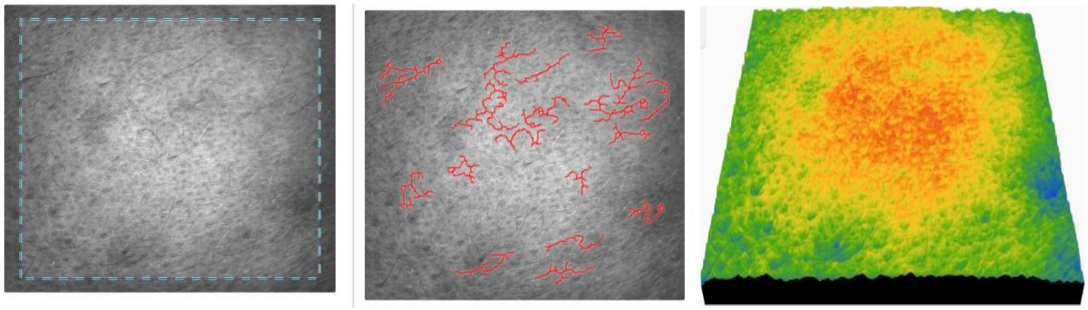

C

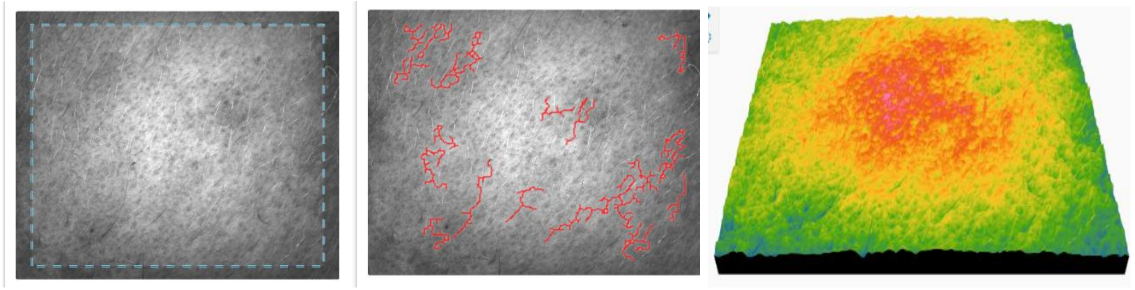

D

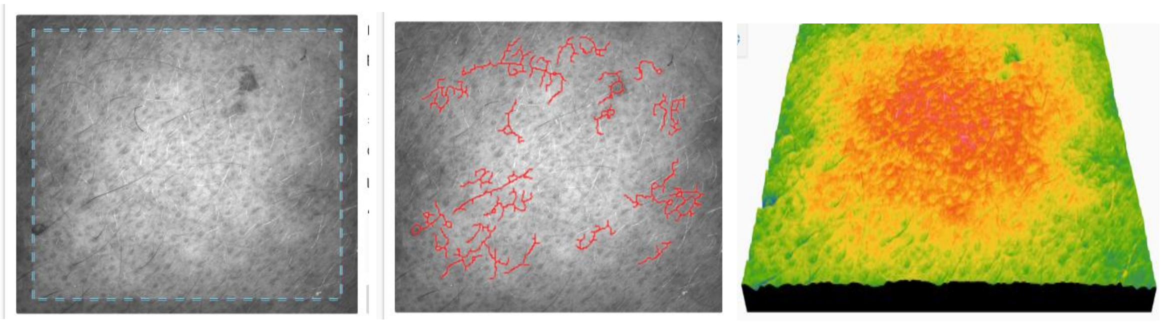

E

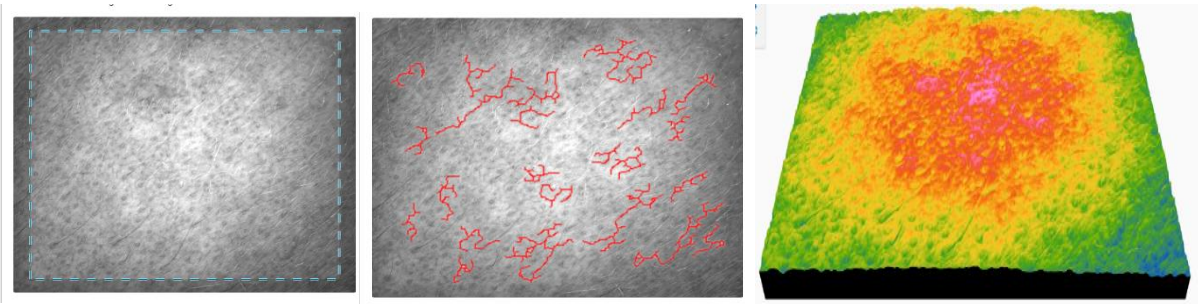

F

Figure S3. The images registered for subject no.26 (age 49) when using ACC: A. before the first application; B. at 30 minutes after the first application; C. after 10 days; D. after 30 days; E. after 60 days; F. after 90 days.

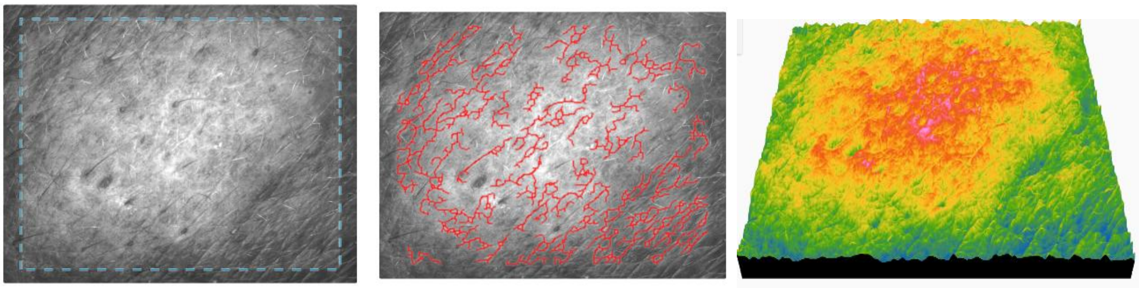

A

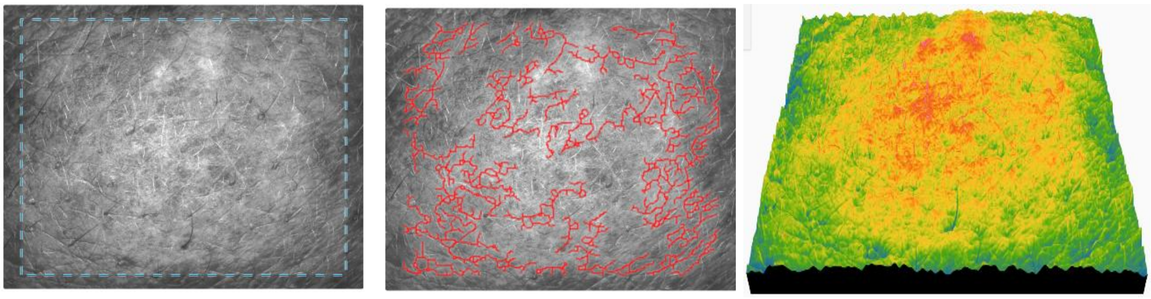

B

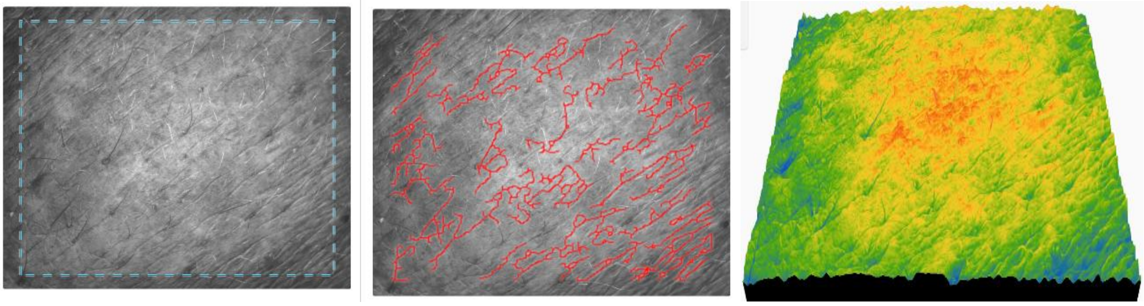

C

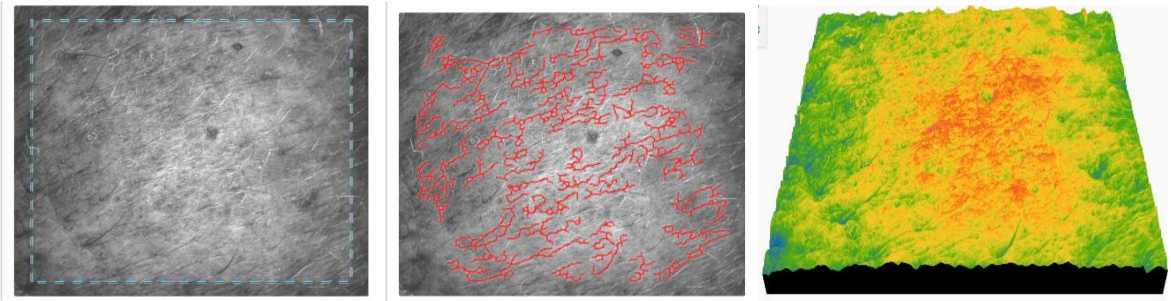

D

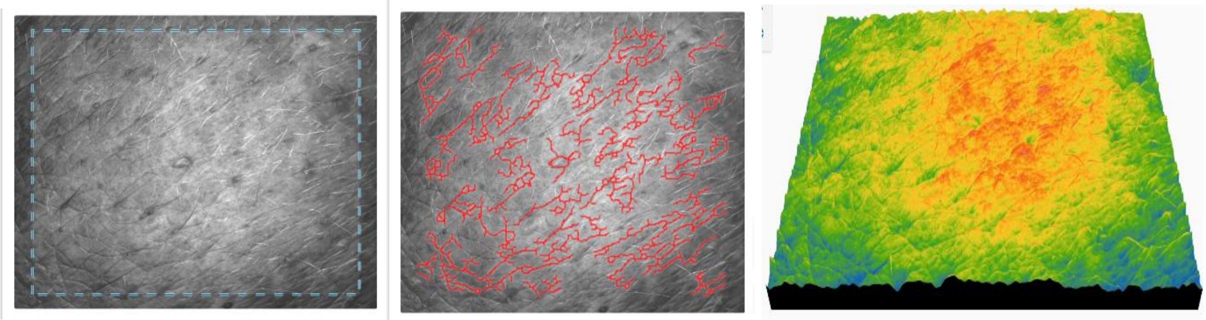

E

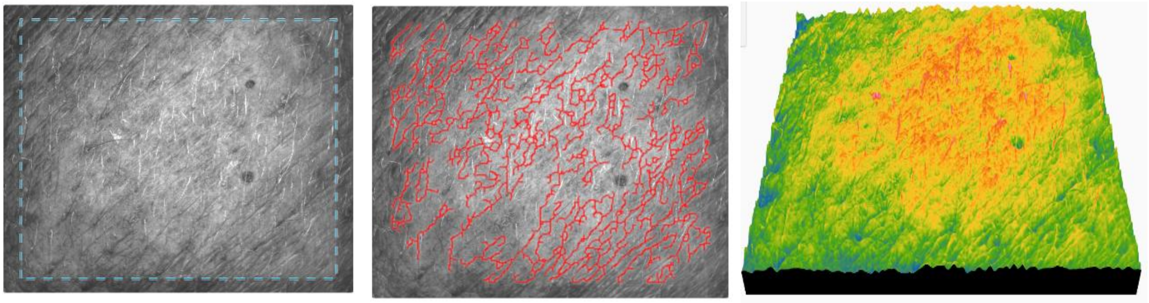

F

Figure S4. The images registered for subject no. 37 (age 48) when using ACC: A. before the first application; B. at 30 minutes after the first application; C. after 10 days; D. after 30 days; E. after 60 days; F. after 90 days.

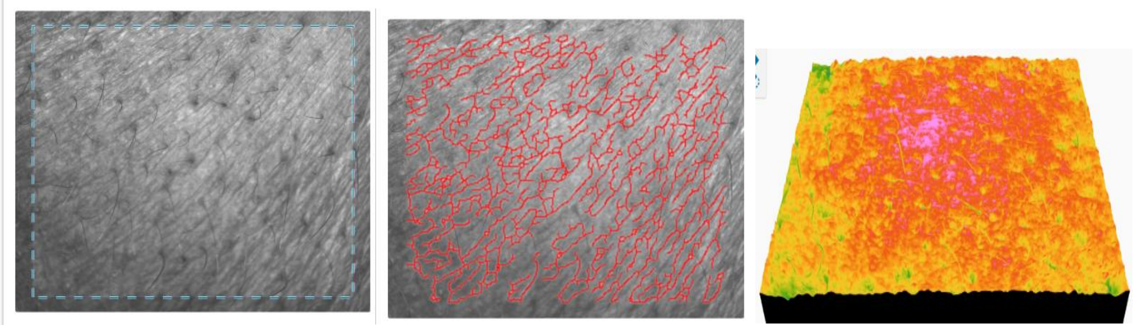

A

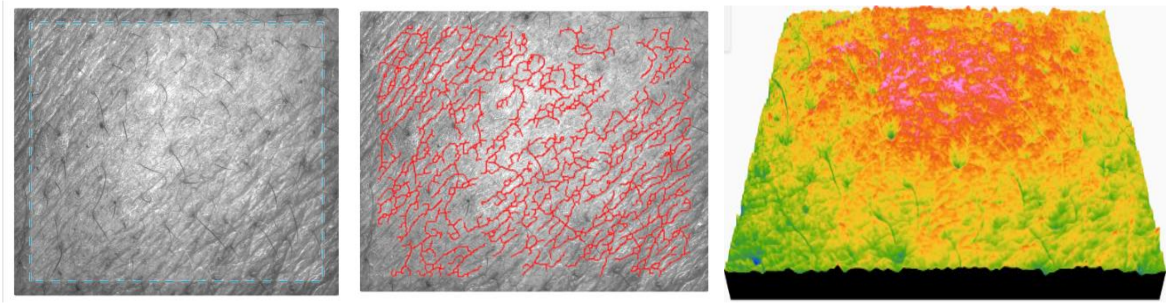

B

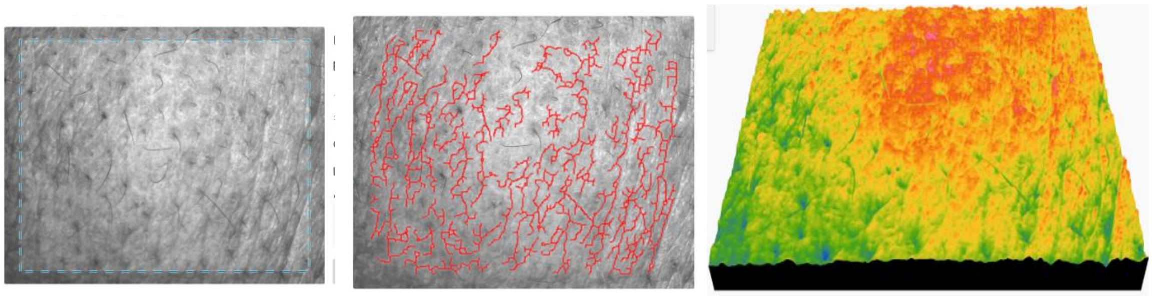

C

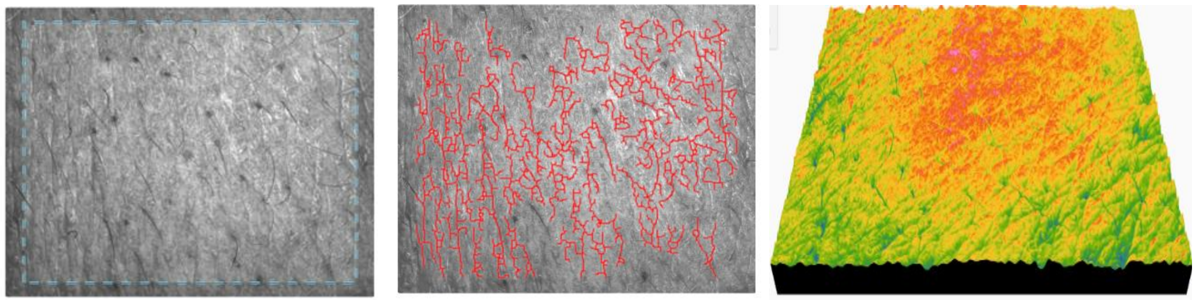

D

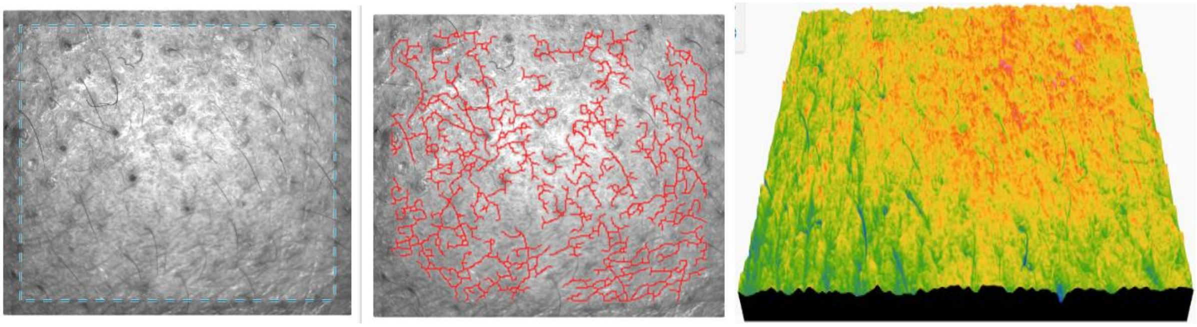

E

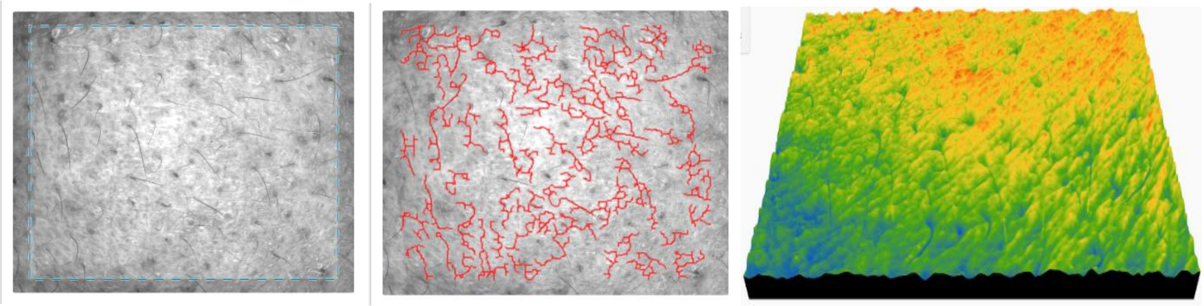

F

Figure S5. The images registered for subject no. 15 (age 50) when using IRC: A. before the first application; B. at 30 minutes after the first application; C. after 10 days; D. after 30 days; E. after 60 days; F. after 90 days.

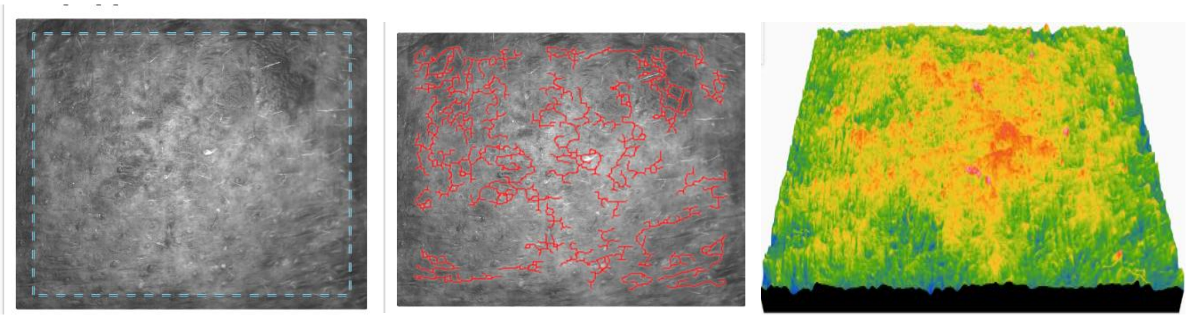

A

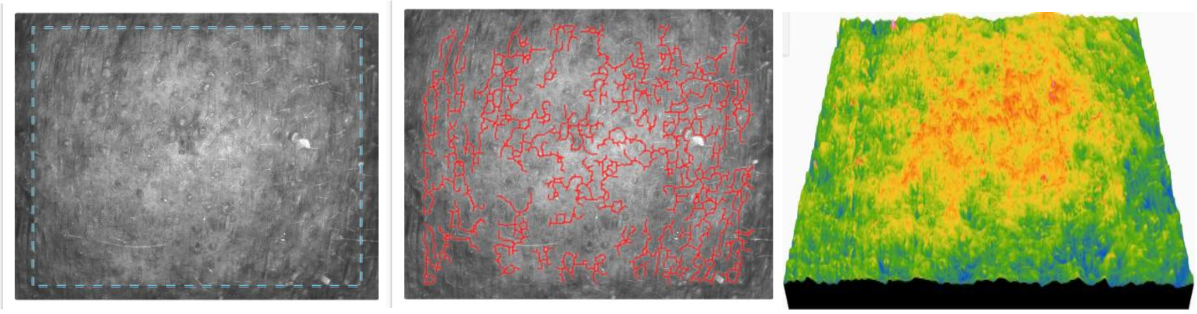

B

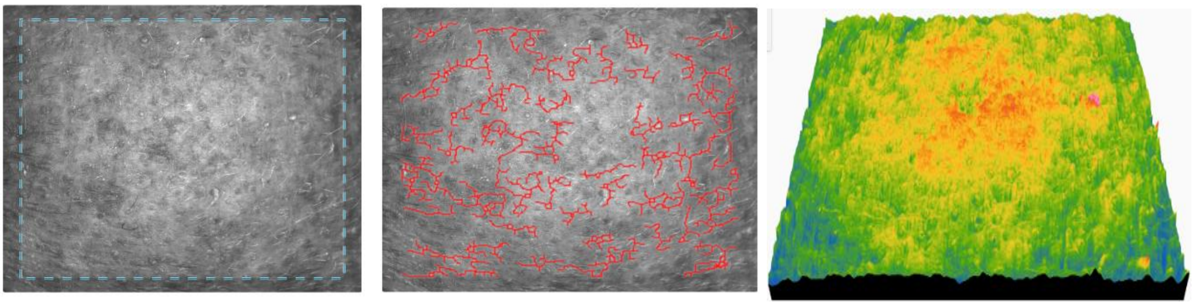

C

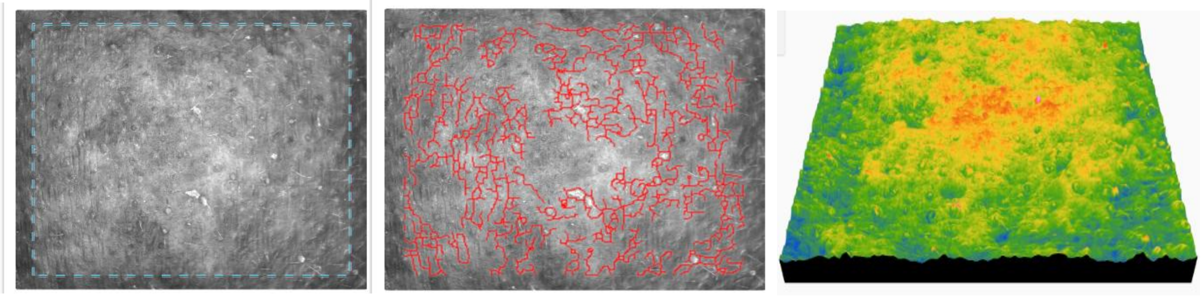

D

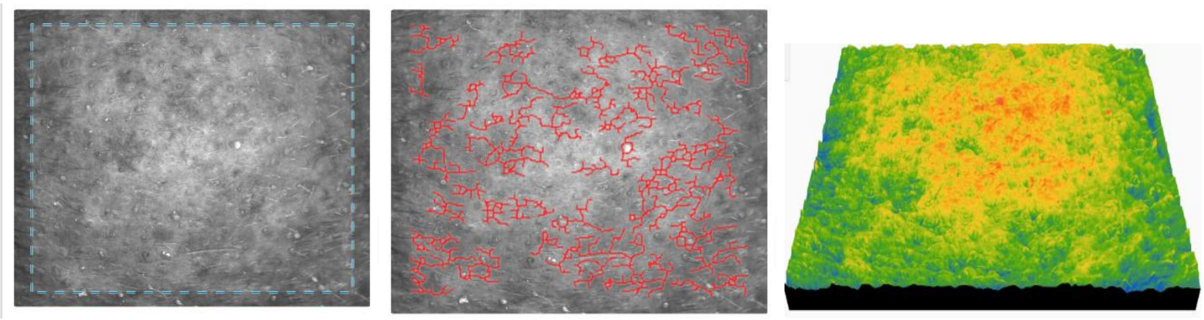

E

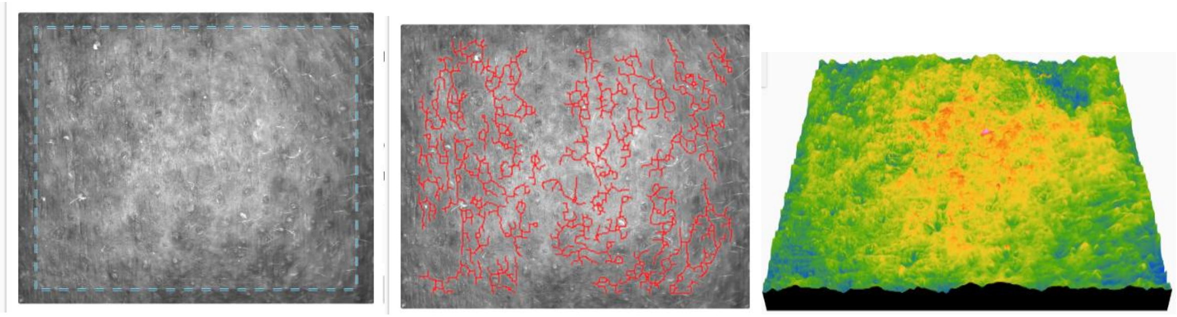

F

Figure S6. The images registered for subject no. 15 (age 50) when using IRC: A. before the first application; B. at 30 minutes after the first application; C. after 10 days; D. after 30 days; E. after 60 days; F. after 90 days.
